# Molecular Mechanisms of Plant Stress Tolerance: From Stress Perception to Phytohormonal Crosstalk and Transcriptional Regulation

**DOI:** 10.3390/cimb48050474

**Published:** 2026-05-02

**Authors:** Sajid Ali, Yong-Sun Moon

**Affiliations:** Department of Horticulture and Life Science, Yeungnam University, Gyeongsan 38541, Republic of Korea

**Keywords:** plant stress tolerance, stress perception, phytohormonal crosstalk, transcriptional regulation, stress memory, signaling networks, transcription factors, crop resilience

## Abstract

In recent years, plant stress biology has moved beyond single-pathway descriptions toward an integrated framework in which stress perception, hormonal control, and gene regulation are tightly interconnected. Early events such as membrane-associated sensing, calcium influx, reactive oxygen species (ROS) generation, and kinase activation converge with phytohormonal networks to shape context-dependent responses. Within this framework, abscisic acid, salicylic acid, jasmonates, ethylene, auxin, cytokinins, gibberellins, brassinosteroids, and strigolactones function not as isolated regulators but as components of a dynamic signaling matrix that balances survival, defense, growth restraint, and recovery. These hormonal signals are ultimately translated into adaptive outcomes through extensive transcriptional and post-transcriptional reprogramming mediated by transcription factors, RNA-based regulators, chromatin remodeling, and stress memory mechanisms. This review synthesizes current understanding of how plants integrate stress perception, phytohormonal crosstalk, and transcriptional regulation to establish stress tolerance. We first examine the molecular basis of stress sensing and early signaling. We then discuss the central functions of major phytohormones and the logic of hormone–hormone interaction networks in coordinating stress adaptation. Next, we analyze transcriptional, post-transcriptional, and epigenetic mechanisms that determine response specificity, intensity, and persistence. We further highlight points of convergence between abiotic and biotic stress responses and discuss how combined stresses challenge traditional single-stress models. Finally, we consider the roles of omics, systems biology, and translational technologies in decoding and engineering stress-resilient phenotypes. By integrating these perspectives, this review presents plant stress tolerance as a multilevel systems property and outlines key priorities for future research aimed at developing climate-resilient crops.

## 1. Introduction

Plants exist in environments where optimal conditions are transient, whereas fluctuations in temperature, water availability, salinity, oxygen status, nutrient supply, and biological attack are persistent realities [1,2]. These environmental pressures shape plant growth, development, reproduction, and survival across both agricultural and natural ecosystems. In crop systems, stress directly compromises yield stability, quality, and resource-use efficiency. In natural ecosystems, it influences species distribution, competitive fitness, and community structure [3,4]. As climatic instability intensifies, plant stress tolerance has become one of the most important themes in contemporary plant biology because it links molecular adaptation with food security and ecological resilience [5]. Plant stress tolerance is not a single trait but an emergent property. It reflects the ability of plants to maintain viability and functional performance under adverse conditions by coordinating physiological, biochemical, and molecular responses across cellular and organismal scales [6]. This coordination begins with rapid perception of stress-associated signals and extends through intracellular signaling, hormone-mediated regulation, transcriptional reprogramming, metabolic adjustment, structural remodeling, and recovery [7,8]. In this sense, tolerance is not merely survival under damage; it also encompasses the capacity to preserve growth, reproduction, and fitness as far as possible despite environmental challenge.

Plant stresses are often classified into abiotic and biotic categories [9]. Abiotic stresses include drought, salinity, heat, cold, flooding, nutrient imbalance, heavy metals, and oxidative stress, all of which disturb water relations, ion homeostasis, membrane integrity, redox balance, photosynthesis, and energy metabolism. Biotic stresses arise from interactions with viruses, bacteria, fungi, oomycetes, nematodes, insects, and parasitic plants, and they trigger molecular recognition, immune activation, defense-associated metabolism, and structural responses [10,11,12]. Although this distinction is useful at the descriptive level, it is increasingly clear that abiotic and biotic stresses cannot be understood as fully separate biological domains. Many of their downstream responses converge on shared signaling currencies such as calcium, reactive oxygen species, mitogen-activated protein kinase cascades, hormone redistribution, and gene regulatory networks [13,14]. Early stress biology was often built on single-stress frameworks in which drought, salinity, cold, heat, immunity, and herbivory were treated as relatively self-contained pathways. That approach was foundational because it led to the discovery of key receptors, signaling modules, hormone networks, and stress-responsive genes [15,16]. However, such reductionist models now appear insufficient for explaining how plants function in real environments, where multiple stresses frequently occur together or in sequence. A plant experiencing drought plus heat, salinity plus oxidative imbalance, or abiotic stress followed by pathogen attack does not simply superimpose single-stress pathways. Instead, it generates unique signaling states and integrated regulatory outputs. This realization has shifted plant stress biology toward a systems perspective [17,18].

Within this systems perspective, phytohormones occupy a central position [19]. Abscisic acid is indispensable in dehydration and osmotic stress signaling, whereas salicylic acid, jasmonates, and ethylene dominate many immune and wound-associated contexts. At the same time, auxin, cytokinins, gibberellins, brassinosteroids, and strigolactones play major roles in developmental plasticity, resource allocation, and recovery [20,21]. These hormones do not function as isolated signals but as interacting regulatory networks that reshape plant priorities under stress. Their biological significance lies not only in their individual actions but also in their crosstalk, because survival, defense, and growth must be coordinated rather than pursued independently. Equally important is the regulatory layer that translates these upstream signals into adaptive gene expression [22]. Stress-responsive transcription factors, promoter architecture, non-coding RNAs, alternative splicing, chromatin remodeling, and transcriptional memory all contribute to the specificity and persistence of plant responses [23,24,25]. These mechanisms determine whether a plant closes stomata, accumulates osmolytes, reinforces cell walls, activates defense metabolism, redirects carbon allocation, or prepares more effectively for subsequent stress [26,27]. Accordingly, stress tolerance should be interpreted as the outcome of coordinated network behavior, where sensory systems, second messengers, hormone pathways, transcriptional regulators, metabolic capacity, and developmental plasticity act together to determine adaptive performance.

This review synthesizes the molecular logic of plant stress tolerance from the earliest phase of perception through hormonal integration and gene regulation. We first discuss stress perception and early signaling. We then examine the principal phytohormones involved in stress adaptation and how their crosstalk coordinates growth–defense decisions. Next, we analyze the transcriptional, post-transcriptional, and epigenetic layers through which stress responses are executed. We then integrate these mechanisms across abiotic and biotic stress biology and consider the roles of omics, systems biology, and translational technologies in building climate-resilient crops. Throughout, the central argument is that plant stress tolerance should be understood as a multilevel, dynamic, and context-dependent property of biological networks rather than the product of isolated genes or pathways.

## 2. Stress Perception and Early Signaling Networks

The first step in stress adaptation is the conversion of environmental disturbance into intracellular information [28]. Plants lack a single universal stress receptor. Instead, stress perception arises from a distributed sensory architecture spanning the plasma membrane, cell wall interface, cytoplasm, and intracellular organelles [29]. This architecture enables cells to detect changes in osmolarity, ion concentration, membrane tension, redox status, temperature, oxygen availability, and the presence of microbial or damage-derived molecules [30]. Because most environmental challenges impose multiple constraints simultaneously, plants rely on combinatorial sensing rather than one-stress–one-receptor logic [28]. A useful conceptual distinction can be made between primary perception and early signal conversion. Primary perception refers to the initial detection of a physicochemical or biological disturbance, whereas early signal conversion refers to the rapid generation of calcium influx, reactive oxygen species, phosphorylation cascades, ion fluxes, and lipid-derived messengers that carry information inward [31]. In many cases, the molecular identity of the true primary sensor remains uncertain, particularly in abiotic stress biology, where perturbations may be sensed through altered cellular states rather than direct ligand–receptor binding. Even so, recent advances have substantially improved mechanistic understanding of stress initiation [32].

The plasma membrane is the principal interface for many stress-associated cues. Receptor-like kinases and related membrane proteins contribute to both environmental sensing and immune activation [33]. Particularly important is the growing recognition that cell wall integrity is itself a major source of stress information. When drought, salinity, mechanical disturbance, or growth inhibition perturbs the wall–membrane continuum, this change can be detected by receptor systems that link extracellular status to intracellular calcium signaling, redox responses, and hormone-regulated adaptation [33]. Such receptors are not merely passive detectors; they act as integrative hubs that connect structural perturbation with downstream regulatory states. Membrane-associated ion channels provide another major component of early sensing [34]. Hyperosmotic stress, mechanosensory responses, touch, hypo-osmotic shock, and temperature fluctuations are all associated with changes in calcium-permeable channel activity [35]. These channels do more than allow ion movement; they generate the first measurable intracellular signatures of stress. Lipids also participate directly in sensory logic, because changes in membrane composition, fluidity, and sphingolipid organization can influence how cells distinguish osmotic, ionic, and redox stress. The membrane should therefore be viewed not as a passive barrier but as an active signaling platform in which proteins, lipids, and the cell wall function together [36].

Temperature and oxygen stress illustrate why perception is best described as distributed. Heat and cold alter membrane fluidity, protein conformation, nucleocytoplasmic dynamics, chromatin organization, and metabolic flux, and these changes are interpreted through partially overlapping mechanisms rather than through a single thermal receptor [34]. Likewise, flooding is sensed not only as reduced oxygen availability but also through rapid shifts in energy charge, mitochondrial function, pH, and redox state [37]. This diversity of perceptual routes helps explain why the earliest phase of stress biology often emerges from perturbed cellular homeostasis rather than from one discrete receptor event [38]. Biotic stress perception is more clearly defined at the receptor level. Plasma membrane pattern-recognition receptors detect pathogen- or damage-associated molecular patterns and activate pattern-triggered immunity [39]. When adapted pathogens deliver effectors to suppress this first layer of defense, intracellular immune receptors recognize effector activity and initiate stronger defense-associated responses [40]. Despite this clearer receptor logic, biotic stress signaling quickly converges with abiotic signaling at the level of calcium, ROS, kinase cascades, hormonal redistribution, and transcriptional control [41]. Thus, specificity is not generated by entirely separate systems, but by different combinations and temporal patterns within shared regulatory infrastructure.

Among early ionic signals, Ca^2+^ is one of the most information-rich. Stress-induced calcium elevations differ in amplitude, duration, oscillatory behavior, propagation pattern, and subcellular location. These “calcium signatures” are decoded by calmodulins, calcium-dependent protein kinases, and CBL–CIPK networks, which regulate transporters, enzymes, transcription factors, and membrane behavior [42]. Ca^2+^ therefore serves not merely as a stress marker but as a structured coding ion through which plants distinguish stimulus identity and intensity [43]. Reactive oxygen species form a second major signaling currency. Although excessive ROS can damage proteins, membranes, and nucleic acids, controlled ROS generation is essential for stress signaling, immune activation, cell wall reinforcement, and systemic communication [44]. Calcium and ROS frequently operate in mutual amplification loops, with calcium activating ROS-producing enzymes and ROS modifying channel activity and receptor behavior. The biological significance of ROS depends on source, concentration, chemical form, and persistence, meaning that ROS function as highly context-dependent signaling molecules rather than generic damage by-products [45].

Gasotransmitters add another important layer to early stress signaling and redox regulation. Nitric oxide (NO) and hydrogen sulfide (H_2_S) are small, diffusible signaling molecules that participate in plant responses to drought, salinity, temperature extremes, heavy metals, pathogen attack, and oxidative stress. Rather than functioning as classical phytohormones, they act as redox-active signaling mediators that modulate protein activity, antioxidant responses, ion transport, and hormone sensitivity. Their effects are closely linked to post-translational modifications of cysteine residues, particularly NO-mediated S-nitrosylation and H_2_S-mediated persulfidation. Through these modifications, NO and H_2_S can alter the catalytic activity, stability, localization, or interaction capacity of signaling proteins, transcription factors, antioxidant enzymes, and hormone-related regulators. NO can also influence chromatin-associated regulation by affecting histone deacetylase activity, thereby linking redox signaling with stress-responsive transcriptional reprogramming. Importantly, NO, H_2_S, and ROS do not act as isolated messengers; they form an interconnected redox network in which each signal can modify the production, scavenging, or downstream effects of the others. This redox-based crosstalk provides a mechanism through which plants convert transient stress-derived chemical signals into changes in protein function and gene expression [46,47].

**Figure 1:**
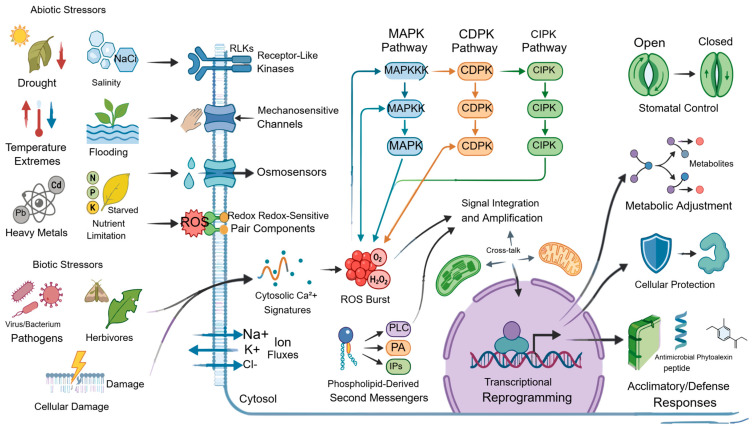
Plant stress perception and early signaling networks linking external stress cues to acclimation and defense responses. Abiotic and biotic stress cues are perceived by membrane-associated receptors and sensors, triggering Ca^2+^ signatures, ROS bursts, ion fluxes, phospholipid-derived messengers, and MAPK, CDPK/CPK, gasotransmitter signaling, and CIPK signaling pathways. These signals are integrated across cellular compartments to activate transcriptional reprogramming, stomatal regulation, metabolic adjustment, cellular protection, and acclimatory or defense responses.

These early signatures are tightly coupled to protein phosphorylation cascades, especially mitogen-activated protein kinases and calcium-dependent protein kinases. Such cascades convert short-lived sensory events into more stable regulatory programs by relaying, filtering, and amplifying signal information [48]. They influence stomatal movement, defense gene activation, metabolic reprogramming, programmed cell death, and recovery processes. Lipid-derived messengers add further complexity by linking membrane perturbation with vesicle trafficking, ion transport, and hormone responsiveness [39]. A defining feature of early stress signaling is integration across space and time. Local perception can trigger systemic calcium waves, ROS waves, electrical signals, and hormonal redistribution, allowing the whole plant to respond to localized damage or environmental change [32]. Specificity emerges not from any one messenger alone but from the combination of signal amplitude, duration, localization, sequence, and tissue context [33]. This principle explains how the same signaling currencies can participate in responses to drought, salinity, heat, wounding, and infection while still producing different physiological outcomes. Figure 1 depicts stress perception as a multilayered continuum extending from external cues to sensory modules, second messengers, and early acclimatory outputs.

## 3. Phytohormonal Control of Plant Stress Responses

Phytohormones are central to plant stress adaptation because they translate early signaling events into coordinated developmental, metabolic, and transcriptional programs [49]. Whereas calcium, ROS, ion fluxes, and phosphorylation cascades dominate the earliest intracellular phase, hormones establish the integrative framework that determines whether the plant prioritizes water conservation, defense activation, tissue protection, growth restraint, architectural remodeling, or recovery. Hormonal regulation is therefore not simply downstream of early signaling; it is the level at which whole-plant adaptive strategy becomes defined [50].

The hormone most closely associated with abiotic stress tolerance is abscisic acid (ABA). ABA accumulates during drought, osmotic stress, salinity, and many temperature-related challenges, and it coordinates stomatal closure, osmotic adjustment, antioxidant defense, membrane stabilization, hydraulic signaling, and large-scale stress-responsive gene expression [51]. The core ABA signaling module centered on PYR/PYL/RCAR receptors, PP2C phosphatases, and SnRK2 kinases provides one of the best-characterized examples of how stress perception is translated into physiological control. Yet ABA is more than a signal for diverse abiotic stresses. It functions as a systems-level regulator that integrates root-to-shoot communication, ion homeostasis, redox status, gasotransmitter-mediated redox signaling, and growth restraint [52]. Under severe stress, the survival advantages of ABA often come with costs to photosynthesis, cell expansion, and biomass accumulation, revealing the central tension between protection and productivity. Salicylic acid (SA), jasmonates (JAs), and ethylene (ET) play equally important roles in defense-centered and mixed stress responses. SA is strongly associated with resistance to biotrophic and hemibiotrophic pathogens and with the establishment of systemic acquired resistance [28,53]. JA is central to responses against herbivores, necrotrophic pathogens, and mechanical damage, and it also contributes to certain abiotic acclimation processes. ET modulates defense, senescence, flooding responses, cell wall remodeling, and developmental plasticity under stress [54]. These three hormones are often introduced through simplified defense categories, but their biological influence is much broader. SA also affects redox homeostasis, JA influences metabolism and long-term resource allocation, and ethylene can reshape root growth, tissue differentiation, and recovery dynamics [55].

Gasotransmitters also participate in the physiological outputs of phytohormone signaling, especially in guard-cell regulation. In ABA-mediated drought responses, H_2_S produced through L-cysteine desulfhydrase activity functions as a component of the guard-cell signaling network and can act upstream of NO during stomatal closure. In Arabidopsis, DES1-dependent H_2_S production is required for full ABA-induced NO accumulation and stomatal closure, indicating that gasotransmitter crosstalk is part of the ABA response module. H_2_S can further strengthen ABA signaling through persulfidation of SnRK2.6/OST1, whereas NO can exert negative feedback on ABA signaling through S-nitrosylation of the same kinase. These examples show that NO and H_2_S can cooperate, compete, or act sequentially depending on the target protein and physiological context. Beyond ABA, NO-mediated S-nitrosylation has been linked with SA-, JA-, auxin-, cytokinin-, ethylene-, and brassinosteroid-related signaling components, supporting the view that gasotransmitters fine-tune hormone networks rather than acting as independent hormone-like regulators [56,57].

Hormones traditionally associated with growth are now recognized as major determinants of stress plasticity. Auxin plays a particularly important role in regulating root architecture, tissue regeneration, cell expansion, and organ patterning under adverse conditions [58]. Stress alters auxin biosynthesis, transport, and signaling, thereby redirecting development rather than simply suppressing it [59]. In drought or salinity, such changes can optimize root exploration, adjust gravitropic behavior, and modify resource acquisition [60]. Cytokinins contribute by balancing growth maintenance with stress survival. Reduced cytokinin signaling often supports conservative survival strategies through restricted shoot growth and altered source–sink relations, but excessive cytokinin decline can accelerate senescence and diminish recovery potential [61]. Conversely, finely tuned cytokinin activity can preserve photosynthetic competence, sustain meristem function, and support post-stress restoration. Their biological role is therefore not simply pro-growth but regulatory, acting at the interface between productivity and resilience. Gibberellins (GAs) and brassinosteroids (BRs) further illustrate how stress adaptation is inseparable from developmental control [62]. Stress commonly suppresses GA signaling, stabilizing DELLA proteins that restrain elongation growth and redirect resources toward survival. However, complete suppression of growth is not always adaptive; recovery, reproduction, and organ maintenance may still require controlled GA activity [63]. Brassinosteroids often help preserve cellular function under stress by improving membrane stability, antioxidant capacity, and metabolic resilience. These examples make clear that growth-related hormones do not uniformly oppose tolerance. Rather, they determine how much growth is sacrificed, redirected, or preserved under particular stress conditions [64].

Strigolactones add another layer of adaptive control by influencing root development, branching, nutrient foraging, and interactions with ABA and auxin. Emerging evidence also points to modulatory roles for peptide signals, melatonin-related pathways, and other non-canonical regulators [65]. Taken together, these findings show that plant stress responses are governed not by a single dominant hormone but by an endocrine network in which multiple pathways cooperate, compete, and recalibrate development in response to environmental demand [66]. A crucial point in any review of hormonal stress biology is that hormone abundance alone rarely predicts biological outcome. The same hormone level can lead to different responses depending on receptor abundance, transport dynamics, conjugation status, tissue sensitivity, and interaction with other pathways [67]. Hormonal function is therefore best understood as a signaling state rather than a concentration. This perspective avoids oversimplified statements and better reflects the reality that plants make adaptive decisions through integrated network configurations. We summarized (Table 1) the main phytohormones involved in stress adaptation, their main stress contexts, signaling components, downstream functions, and relationships to growth–defense balancing.

## 4. Phytohormonal Crosstalk in Coordinating Stress Tolerance

If individual hormones define regulatory capacities, hormonal crosstalk defines adaptive logic. Stress responses are not determined by the action of one hormone alone but by the relationships among multiple hormonal pathways. Crosstalk allows plants to adjust priorities dynamically rather than committing to rigid, preprogrammed outputs [84]. It is mediated through reciprocal effects on biosynthesis and catabolism, modulation of receptor sensitivity, competition or cooperation among transcription factors, and convergence on shared downstream genes and metabolic processes [85]. One of the most biologically important interactions is between ABA and SA. ABA is indispensable for dehydration and osmotic stress survival, yet strong ABA signaling can attenuate certain immune outputs, especially those associated with SA-dependent defense [86]. This antagonism helps explain why abiotic stress can alter pathogen outcomes and why plants sometimes become more vulnerable to disease under drought or salinity. However, the relationship is neither constant nor absolute. Depending on timing, tissue context, pathogen identity, and signal amplitude, ABA and SA may coexist, alternate in dominance, or partially override one another [87]. Their interaction is therefore best understood as a context-sensitive axis that determines how strongly the plant commits to abiotic conservation versus immune defense.

The JA–ET relationship is often more cooperative, particularly in wound signaling, herbivory, and defense against necrotrophs. Through interactions involving JAZ repressors, MYC factors, EIN3/EIL proteins, and ERF transcription factors, JA and ET coordinate defense gene expression, tissue remodeling, and metabolic reallocation. Even so, their synergy is not unconditional [88]. Developmental stage, nutrient status, and background ABA or SA signaling can alter the strength and outcome of this partnership. The classical SA–JA antagonism remains a useful framework, especially in distinguishing defense programs against biotrophic versus necrotrophic attackers [89]. Yet current understanding is more nuanced than the older binary model. Plants may deploy SA- and JA-centered responses in different tissues, in sequential phases, or in partially overlapping domains. Such flexibility is essential because natural stress scenarios rarely involve one attacker or one signaling demand. The value of crosstalk lies precisely in allowing plants to partition and rebalance defense functions rather than simply turning one pathway off and another on [75].

A major consequence of hormonal crosstalk is the regulation of growth–defense trade-offs. Stress tolerance often requires reduced growth so that resources can be redirected toward osmoprotection, detoxification, repair, and defense [90]. The GA–DELLA–JA module is a classic example: GA promotes growth, DELLA proteins restrain it under adverse conditions, and JA reinforces defense-oriented priorities [91]. Similarly, ABA and auxin interact to reshape root architecture during drought or salinity, not by abolishing development, but by redirecting it toward water acquisition and survival. Cytokinin–ABA interactions influence whether shoot tissues remain metabolically active or shift into conservation and senescence-associated programs [92]. Another important feature of crosstalk is spatial prioritization. Plants do not necessarily respond uniformly across all organs. Under stress, roots, young leaves, mature leaves, reproductive tissues, and meristems may each adopt distinct hormonal states. Such partitioning allows one region to enter strong conservation mode while another remains developmentally active [93]. Hormonal transport, local biosynthesis, receptor sensitivity, and tissue-specific transcriptional networks all contribute to this spatial differentiation.

**Figure 2:**
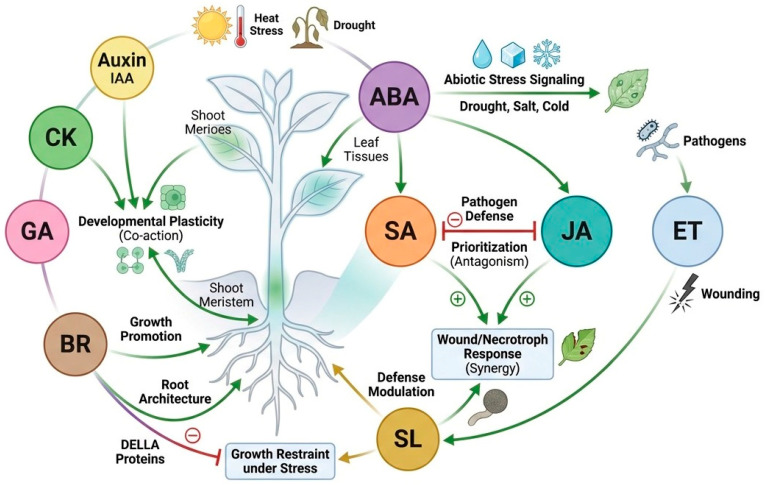
Network model of phytohormonal crosstalk during plant stress adaptation. Conceptual diagram showing the synergistic and antagonistic interactions among major phytohormones during plant stress adaptation. ABA, SA, JA, and ET act as central signaling hubs and interact with auxin, cytokinins, gibberellins, brassinosteroids, and strigolactones to balance growth, development, and defense. The figure highlights ABA-mediated abiotic stress signaling, SA–JA antagonism in defense prioritization, JA–ET synergism in wound and necrotroph responses, GA–DELLA-associated growth restraint under stress, and auxin–cytokinin coordination of developmental plasticity. Overall, the network illustrates how phytohormonal crosstalk fine-tunes plant responses according to stress type and physiological context.

Crosstalk becomes even more critical under combined and sequential stresses. Plants facing drought plus heat, flooding plus salinity, or abiotic stress followed by infection cannot simply sum single-stress responses [94]. Instead, hormones establish a hierarchy of priorities based on urgency, tissue vulnerability, and anticipated recovery. Prior stress exposure can also sensitize or dampen later responses, creating memory-like behavior within hormonal networks. This is one reason why single-hormone engineering often produces inconsistent field outcomes [95]. Manipulation of a single pathway may improve tolerance to one challenge while weakening resistance to another. A more robust strategy may be to target integrative nodes within the crosstalk network that preserve flexibility while reducing excessive trade-off costs [96]. Overall, hormonal crosstalk is the regulatory layer that converts hormone presence into adaptive decision-making. It coordinates survival, defense, development, and recovery across both space and time. Importantly, hormonal hierarchy under combined or sequential stress is not fixed. The dominant regulatory pathway may shift according to stress order, duration, intensity, tissue sensitivity, and developmental stage, allowing plants to prioritize water conservation, immune defense, growth restraint, or recovery according to the most urgent physiological demand. Figure 2 therefore shows the major phytohormones as interacting hubs, linked by synergistic and antagonistic relationships and organized around growth–defense balance and combined-stress adaptation.

## 5. Transcriptional and Post-Transcriptional Regulation of Stress Responses

The transition from stress signaling to adaptive phenotype depends on whether upstream signals are translated into precise, timely, and energetically sustainable changes in gene expression. Therefore, transcriptional and post-transcriptional regulation represent the execution layer through which perception, second messengers, and hormone networks are converted into functional stress responses. The adaptive outcome of stress signaling depends on the extent to which the plant can reprogram gene expression. Stress tolerance therefore requires coordinated transcriptional reprogramming in which plants suppress many growth-associated processes while activating protective, defensive, and acclimatory programs [97]. This transition is not passive. It is actively orchestrated by transcription factors, promoter architecture, chromatin state, RNA processing, and translational control [98]. Several transcription factor families repeatedly emerge as core regulators of plant stress responses. AP2/ERF-DREB proteins are central to dehydration, salinity, cold, and many mixed stress responses. bZIP factors, particularly ABF/AREB-type regulators, are prominent in ABA-mediated control of osmotic adaptation [99]. NAC transcription factors contribute to survival-oriented adjustment, resource reallocation, senescence-linked plasticity, and long-term stress endurance. WRKY proteins are especially important in defense signaling and redox-associated regulation, whereas MYB and bHLH proteins connect stress responses with secondary metabolism, developmental control, and hormone integration. Heat shock factors and zinc finger proteins add further specialization in proteostasis, oxidative balance, and signal fine-tuning [100].

These transcription factors should not be viewed as isolated master switches. Their biological effect depends on expression level, post-translational modification, interaction partners, chromatin accessibility, and competition for promoter occupancy [101]. A factor that enhances tolerance in one tissue or condition may behave differently in another because the surrounding regulatory network has changed. This is why overexpression studies, although informative, do not always translate directly into stable field performance [102]. Stress-responsive transcription factors are more accurately described as network nodes whose function is defined by context. Equally important is the cis-regulatory architecture of target genes [103]. Stress-responsive promoters contain combinations of motifs such as ABREs, DRE/CRT elements, W-boxes, GCC-boxes, and heat shock elements. These motifs enable genes to integrate signals from ABA, osmotic sensing, WRKY-mediated defense, ERF-associated stress responses, and proteostasis-related pathways [104]. As a result, many genes can respond to more than one stimulus, and their exact expression pattern depends on which cis-elements are present, which transcription factors are available, and how chromatin structure shapes accessibility.

Beyond transcription initiation, plant stress responses are refined by multiple post-transcriptional mechanisms. MicroRNAs, small interfering RNAs, and long non-coding RNAs modulate transcript stability, translational efficiency, chromatin interactions, and network connectivity [105]. Stress-responsive miRNAs frequently target transcription factors or signaling proteins, thereby linking developmental regulation with defense and acclimation. Long non-coding RNAs can act as scaffolds, decoys, guides, or chromatin-associated regulators and are increasingly recognized as contributors to stress-specific regulatory precision [106]. Alternative splicing greatly expands regulatory flexibility. Many plant genes produce multiple transcript isoforms, and stress can alter splicing patterns in kinases, transporters, transcription factors, and metabolic regulators [107]. These isoform changes influence protein domain structure, subcellular localization, binding specificity, and transcript stability. Consequently, transcript abundance alone often provides an incomplete picture of regulatory output. Additional layers such as selective translation, RNA storage, and RNA decay help plants prioritize essential proteins during acute stress and recovery [108].

Epigenetic regulation gives plant stress responses a temporal dimension. DNA methylation, histone modifications, chromatin remodeling, and RNA-directed silencing influence gene accessibility and responsiveness [109]. In some cases, stress leaves a transient or semi-stable memory that alters the speed or magnitude of later responses. Such memory may involve persistent chromatin marks, altered transcription factor recruitment, or primed hormone–metabolism states [110]. Although not every stress generates durable memory, these mechanisms are central to understanding how plants acclimate to repeated environmental challenges. Stress regulation is also deeply linked to metabolic economy [111]. Protective outputs such as osmolyte accumulation, antioxidant regeneration, cell wall reinforcement, and defense metabolite production require carbon, nitrogen, sulfur, and energy investment. Transcriptional and post-transcriptional regulation therefore does not simply control defense genes; it also manages resource allocation [112,113]. A plant that signals strongly but fails to sustain metabolic supply may still collapse under prolonged stress. For this reason, regulatory success must be evaluated not only by gene expression but by its metabolic consequences.

Taken together, transcriptional and post-transcriptional mechanisms transform early signaling and hormonal states into adaptive physiological programs. They determine which genes respond, how strongly they respond, in which cells they respond, and whether the response is transient, sustained, or primed for future challenge. In Table 2, we summarized the major transcription factor families, RNA-based regulators, upstream signals, representative target processes, and adaptive outputs involved in plant stress tolerance.

## 6. Integrated Molecular Mechanisms of Abiotic and Biotic Stress Tolerance

One of the clearest conclusions from modern plant stress research is that abiotic and biotic stress responses are deeply interconnected. Distinct stresses enter the system through different sensory routes, yet they converge rapidly on shared second messengers, hormone networks [127], and gene regulatory modules. This does not mean that all stresses become biologically equivalent [132]. Rather, plants preserve stress identity through differences in signal timing, amplitude, localization, and network context, while still relying on a common regulatory infrastructure. Across abiotic stresses such as drought, salinity, heat, and cold, plants repeatedly mobilize a shared toolkit consisting of osmotic adjustment, ion homeostasis, ROS detoxification, proteostasis, membrane stabilization, and developmental reallocation. ABA often dominates these responses, but ethylene, jasmonates, brassinosteroids, and growth-regulating hormones strongly modify the outcome [133,134]. Transcription factors that respond to one abiotic stress frequently participate in others, which helps explain cross-protective acclimation and the existence of core stress-responsive gene sets. Nevertheless, the relative weighting of these components varies with genotype, developmental stage, and stress combination, so there is no single universal abiotic tolerance pathway [135,136].

Biotic stress responses are equally integrated with general stress physiology. Immune receptors initiate pathogen recognition, but downstream resistance depends on calcium signaling, ROS dynamics, hormone crosstalk, transcriptional reprogramming, and metabolic redistribution [137]. Herbivory combines mechanical damage, electrical signaling, wound-associated hormone responses, defense metabolism, and developmental plasticity. Even classical immunity cannot be understood apart from water status, redox balance, energy availability, and tissue-specific growth decisions [138]. Defense is therefore embedded within the broader logic of plant stress adaptation rather than standing apart from it. The importance of this integration becomes most apparent under combined stress. A plant experiencing drought and pathogen attack, for example, must maintain ABA-driven water conservation while avoiding excessive suppression of SA- or JA-dependent defense [139]. Heat may improve tolerance to one process while undermining another by destabilizing proteins or altering immune signaling thresholds. Flooding shifts ethylene dynamics in ways that affect both acclimation and disease outcome. In these cases, plants often express unique genes and regulatory states that are not strongly induced by either single stress alone. This non-additivity is one of the strongest arguments against treating abiotic and biotic stress biology as separate fields [140].

Another key point is that metabolism acts as both output and determinant of integrated tolerance. Osmolytes, antioxidants, defense metabolites, and membrane lipids do not simply appear as endpoints of signaling; their synthesis and turnover shape cellular capacity to sustain the response [141]. Carbon allocation, nitrogen remobilization, mitochondrial activity, and photosynthetic adjustment determine whether signaling can be maintained long enough to preserve viability and performance [142]. Thus, integrated stress tolerance cannot be explained solely by signaling pathways; it must also be understood through metabolic capacity and resource economy [143].

**Figure 3:**
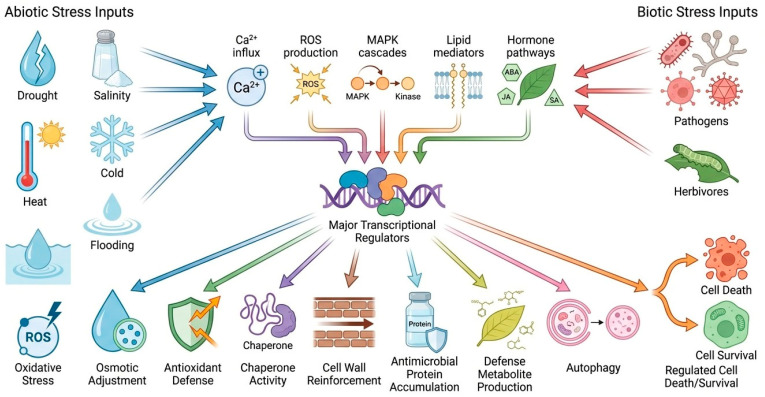
Integrated molecular framework linking abiotic and biotic stress tolerance. Abiotic and biotic stress cues activate overlapping signaling modules, including Ca^2+^ influx, ROS production, MAPK cascades, lipid signaling, and hormone pathways. These upstream signals converge on major transcriptional regulators and trigger downstream protective responses such as osmotic adjustment, antioxidant defense, chaperone activity, cell wall reinforcement, antimicrobial protein accumulation, defense metabolite production, autophagy, and regulated cell death or survival.

The translational implications are substantial. Improving tolerance to one stress may unintentionally weaken another trait if the intervention disturbs shared signaling or hormonal hubs [144]. Conversely, targeting integrative regulators can sometimes generate broader resilience if trade-off costs are carefully managed. This is why network-informed intervention is generally more promising than simple enhancement of a single pathway [145]. A framework is demonstrated in Figure 3, showing abiotic and biotic stresses entering through distinct sensory routes, converging on shared signaling and hormonal hubs, and diverging again into output pathways such as osmoprotection, antioxidant defense, antimicrobial metabolism, cell wall reinforcement, and regulated survival or containment responses.

## 7. Emerging Omics Approaches and Translational Perspectives

The rapid development of omics technologies has transformed plant stress biology from a gene-centered discipline into a multiscale systems science. Genomics, transcriptomics, proteomics, metabolomics, epigenomics, and phenomics now make it possible to investigate stress adaptation across molecular and organismal levels [146]. This expansion has changed not only how much information can be collected, but also the kinds of questions that can be asked. Researchers can now reconstruct regulatory networks, identify pathway bottlenecks, discover biomarkers, and prioritize targets for breeding or genome editing with far greater precision than before. Transcriptomics remains the most widely used platform for mapping stress-responsive genes and co-expression modules [147]. It provides a broad view of transcriptional shifts under different stress conditions and is especially useful for identifying candidate regulators. Proteomics adds functional depth by revealing changes in signaling proteins, enzymes, chaperones, and post-translational modification states that are not always predictable from RNA abundance [128,148]. Metabolomics captures the biochemical outputs of stress adaptation, including osmolytes, antioxidants, hormones, and defense-related secondary metabolites. Epigenomic profiling reveals DNA methylation patterns, histone states, and chromatin accessibility associated with response plasticity and memory [149]. When integrated, these layers provide a far more complete understanding of stress adaptation than any one dataset alone.

A major recent advance is the rise in single-cell and spatial omics. Bulk tissue analyses obscure cellular heterogeneity, yet roots, mesophyll, guard cells, vascular tissues, meristems, and reproductive organs often respond differently to the same stress [138]. Single-cell transcriptomics can identify cell-type-specific regulators and response states, while spatial transcriptomics retain positional information and local signaling context. These approaches are beginning to reveal how stress propagates across tissues, how developmental gradients affect responsiveness, and which cell populations are most important for tolerance [150]. Such resolution is especially valuable for crops, where tissue-specific protection may determine reproductive success or yield stability. At the phenotypic level, high-throughput phenotyping provides the crucial bridge between molecular discovery and whole-plant performance. Imaging and sensor-based platforms can quantify growth, water status, canopy temperature, chlorophyll dynamics, senescence, lesion development, and other traits across large populations [151]. This makes it possible to connect molecular features with physiologically meaningful outcomes and to evaluate genotype-by-environment interactions more realistically. Without such phenotypic validation, even the most sophisticated omics discovery remains only partially informative [152].

As datasets grow in size and complexity, computational integration becomes indispensable. Gene regulatory network inference, multi-omics integration pipelines, machine learning, and explainable artificial intelligence are increasingly used to identify regulatory hubs, infer causal relationships, and predict stress-associated phenotypes [153]. AI also plays a major role in image-based stress detection, phenotyping automation, and classification of complex stress signatures. These tools are helping shift the field from descriptive analysis toward predictive plant stress biology [154]. The translational value of these approaches is especially evident in molecular breeding and genome editing. Omics-guided target discovery can identify robust candidates for CRISPR-based intervention, marker development, or genomic selection. Yet translation to the field remains difficult [155]. Controlled-environment datasets often lose predictive power under variable agricultural settings, where multiple stresses co-occur and environmental noise is high. Standardization of data acquisition, integration of molecular and phenotypic scales, and strong field-based validation remain major bottlenecks [156].

Temporal integration across scales is also becoming increasingly important. Early signaling unfolds within seconds to minutes, transcriptional shifts develop over minutes to hours, metabolic remodeling persists for days, and developmental or memory effects may influence entire life cycles [157]. Omics platforms that capture only one timescale risk missing the continuity between immediate signaling and long-term phenotypes. The most informative studies are therefore those that explicitly connect rapid molecular dynamics with later agronomic performance [158]. A particularly urgent future priority is the creation of mechanistically informed breeding frameworks. Traditional breeding captures stress adaptation phenotypically, whereas molecular biology often identifies regulators without testing their agronomic stability [159]. The next phase should merge these strengths by combining network-based markers, physiology-informed genomic selection, and genome editing guided by validated regulatory modules rather than isolated genes. This approach is more likely to produce durable gains in resilience because it reflects the multicomponent nature of stress tolerance. In Table 3, we summarized the major omics and systems-level approaches, their biological scope, translational potential, and current limitations.

## 8. Challenges, Knowledge Gaps, and Future Perspectives

Despite substantial progress, several major challenges continue to limit the translation of plant stress biology into durable crop improvement. The first is the complexity and redundancy of stress-regulatory networks [173]. Many genes belong to large families, several hormones influence the same processes, and multiple pathways can compensate for one another. Such redundancy increases biological robustness but complicates mechanistic interpretation and target prioritization [174]. Regulators that appear decisive in one genotype or condition may show weaker, more conditional, or pleiotropic effects in another. A second major challenge is the continued dominance of single-stress experiments [175]. These studies remain valuable for mechanistic dissection, but they do not adequately represent the environments in which crops grow. In the field, plants commonly encounter combinations and sequences of drought, heat, salinity, nutrient limitation, pathogen attack, and herbivory. Combined stresses often generate unique signaling and transcriptomic states, meaning that conclusions drawn from isolated stress conditions may not generalize [175]. Future stress research must increasingly incorporate fluctuating intensity, recovery phases, developmental timing, and sequential stress exposure [176].

The field also remains heavily dependent on a small number of model species, especially Arabidopsis [177]. Model plants have provided indispensable insights into signaling, hormone biology, gene regulation, and chromatin dynamics, but crop species differ in genome structure, developmental architecture, lifespan, reproductive biology, and ecological adaptation [178,179]. Some regulatory modules are well conserved, whereas others are rewired or weighted differently. Stronger cross-species validation is therefore essential if mechanistic findings are to translate into agronomically meaningful applications [180]. A further limitation is inadequate spatiotemporal resolution. Stress responses are dynamic and cell-type-specific, yet many studies still rely on bulk tissues sampled at limited time points. High-resolution approaches such as single-cell and spatial omics are beginning to address this gap, but they remain underused in many crop systems [181]. Similarly, the persistent lab-to-field gap continues to hinder application. Traits that appear promising in growth chambers or controlled greenhouse studies do not always remain advantageous under fluctuating field environments, where soil heterogeneity, management practices, canopy interactions, and genotype-by-environment effects strongly influence phenotype [182].

The role of the plant microbiome represents another major knowledge gap. Rhizosphere and endosphere communities influence nutrient acquisition, hormone balance, disease resistance, and tolerance to abiotic stress [183]. However, their effects are highly context dependent, varying with soil type, climate, genotype, and agricultural management. Future models of stress tolerance must therefore incorporate genotype × environment × microbiome interactions rather than treating the plant as an isolated organism [184].

**Figure 4:**
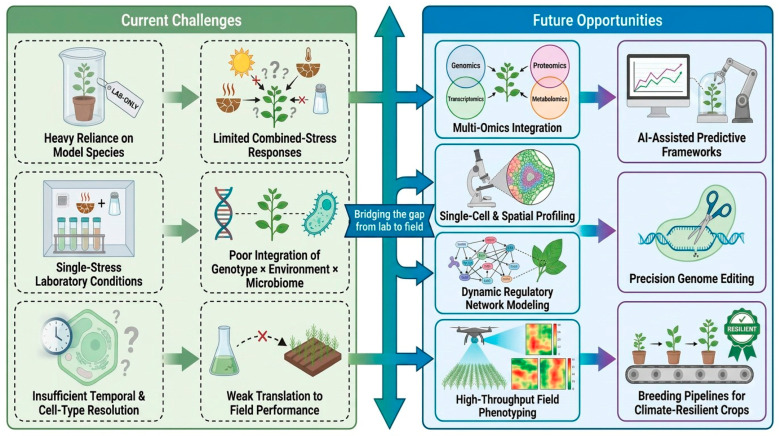
Current challenges and future directions in molecular research on plant stress tolerance. Conceptual roadmap highlighting the main limitations in plant stress research, including reliance on model species, single-stress laboratory conditions, limited resolution of combined and context-dependent responses, poor integration of genotype × environment × microbiome interactions, and weak translation to field performance. It also outlines emerging opportunities, such as multi-omics integration, single-cell and spatial profiling, dynamic regulatory network modeling, high-throughput field phenotyping, AI-assisted predictive frameworks, precision genome editing, and breeding strategies for climate-resilient crops. Elements are represented schematically for conceptual illustration rather than detailed molecular or biochemical mechanisms.

Several future directions follow from these limitations. First, plant stress biology must move toward multiscale integration, combining signaling, hormones, gene regulation, metabolism, phenotype, and environmental context in unified analytical frameworks [185]. Second, more work is needed on combined and sequential stress biology, particularly under conditions that resemble real cropping systems [186]. Third, candidate targets for breeding or engineering should be selected not simply because they are highly induced, but because they occupy robust positions in regulatory networks and show stable phenotypic relevance across conditions [187]. Fourth, molecular discoveries must be paired with high-throughput phenotyping and rigorous field validation [188]. Fifth, microbiome-aware and ecosystem-aware perspectives should become part of mainstream crop resilience research [189]. Ultimately, the field is shifting from reductionist pathway descriptions toward a predictive, systems-based science of plant stress adaptation. Future success will depend on building integrative pipelines that link mechanistic understanding with phenotypic performance under realistic environmental variation. Figure 4 demonstrates this transition as a roadmap from current bottlenecks, including single-stress bias, model-species dependence, low spatiotemporal resolution, and lab-to-field gaps, toward multi-omics integration, spatial and single-cell technologies, AI-assisted prediction, field phenotyping, microbiome-informed biology, and climate-resilient crop design.

## 9. Conclusions

Plant stress tolerance is best understood as a systems-level property that emerges from the integration of stress perception, early signaling, hormonal coordination, and gene regulation. Plants do not survive environmental adversity through isolated pathways. Instead, they deploy interconnected sensory modules, second messengers, kinase cascades, hormone networks, transcription factors, RNA-based regulators, chromatin mechanisms, and metabolic adjustments that together define adaptive capacity. A major theme of this review is that the traditional separation of abiotic and biotic stress biology is increasingly inadequate. Distinct stresses enter through different sensory routes, but they rapidly converge on shared signaling currencies and regulatory hubs, while combined and sequential stresses frequently generate unique, non-additive outcomes. This convergence explains both the flexibility and the complexity of plant stress responses. It also clarifies why manipulation of one regulatory component can improve one trait yet compromise another if it disrupts a shared adaptive node. Phytohormonal crosstalk and transcriptional regulation are the principal integrative layers. Hormones act as dynamic network components rather than isolated signals, and gene regulation determines how these network states are translated into tissue-specific, time-dependent, and stress-specific outputs. Post-transcriptional control, alternative splicing, epigenetic modulation, and stress memory further expand the range and durability of plant adaptive responses. The future of plant stress biology lies in integrative and predictive frameworks. Omics, single-cell and spatial technologies, AI-enabled phenotyping, and genome editing offer unprecedented opportunities to connect molecular mechanisms with trait improvement. Yet their value will depend on realistic multi-stress experimentation, crop-based validation, and field-relevant prediction. Progress in climate-resilient agriculture will therefore require not the search for one universal master regulator, but the capacity to understand and engineer the regulatory networks that allow plants to adapt across changing environments.

## Figures and Tables

**Table 1:** Major phytohormones and their molecular functions in plant stress tolerance. This table summarizes the principal phytohormones involved in plant adaptation to environmental stress, highlighting their predominant stress contexts, major signaling components, core molecular functions, representative downstream responses, and broader relevance to growth–defense balancing. While some hormones are classically associated with either abiotic or biotic stress, increasing evidence indicates that their functions are highly context-dependent and integrated into broader signaling networks.

Phytohormone	Major Stress Contexts	Core Biosynthesis/Signaling Components	Principal Molecular Functions	Representative Downstream Responses	Growth/Defense Trade-Off Relevance
Abscisic acid (ABA)	Drought, salinity, osmotic stress, cold, heat, flooding recovery	NCED biosynthesis enzymes; PYR/PYL/RCAR receptors; PP2Cs; SnRK2s; ABF/AREB transcription factors	Central regulator of abiotic stress signaling; coordinates water conservation, osmotic adjustment, and stress-inducible gene expression	Stomatal closure, LEA protein accumulation, osmoprotectant biosynthesis, ROS detoxification, root architecture modulation	Strongly promotes survival under stress, often at the cost of growth and photosynthetic activity [68,69]
Salicylic acid (SA)	Biotrophic and hemibiotrophic pathogen attack, systemic acquired resistance, oxidative stress	ICS and PAL pathways; NPR1/NPR3/NPR4; TGA transcription factors; PR gene activation	Mediates immune signaling, redox-sensitive defense activation, and systemic resistance	PR protein expression, defense metabolite accumulation, local and systemic immunity, redox homeostasis	Prioritizes defense over growth when pathogen pressure is high; often antagonistic to JA-dependent responses [70,71]
Jasmonates (JA/JA-Ile)	Herbivory, wounding, necrotrophic pathogens, mechanical stress, some abiotic stresses	LOX-AOS-AOC-OPR biosynthesis pathway; COI1 receptor; JAZ repressors; MYC transcription factors	Controls wound responses, anti-herbivore defense, defense metabolite production, and stress acclimation	Proteinase inhibitor induction, secondary metabolite biosynthesis, defense gene activation, growth inhibition under prolonged stress	Promotes defense investment and resource reallocation, frequently restraining vegetative growth [69,72,73]
Ethylene (ET)	Flooding, pathogen attack, wounding, salinity, senescence, mechanical impedance	ACS and ACO enzymes; ETR receptors; CTR1; EIN2; EIN3/EIL1; ERFs	Regulates stress acclimation, senescence, cell wall remodeling, and defense-related transcription	Aerenchyma formation, pathogen-responsive gene induction, modulation of root growth, interaction with JA signaling	Can either restrain or support growth depending on developmental stage and stress type; major regulator of plasticity [74]
Auxin (IAA)	Drought adaptation, salinity, shade-associated stress, wound repair, developmental adjustment under stress	TAA/YUCCA biosynthesis; TIR1/AFB receptors; Aux/IAA repressors; ARFs	Coordinates developmental plasticity, especially root system remodeling and tissue regeneration	Lateral root modulation, tropic growth changes, vascular differentiation, stress-induced architectural adjustment	Maintains growth potential under stress but is frequently repressed or redistributed to favor survival [75,76]
Cytokinins (CKs)	Nutrient stress, drought, senescence regulation, recovery after stress	IPT biosynthesis enzymes; AHK receptors; AHP phosphotransfer proteins; ARR regulators	Regulate cell division, nutrient allocation, meristem activity, and delay senescence	Shoot growth maintenance, chlorophyll retention, source–sink modulation, altered root-to-shoot balance	Often antagonistic to ABA; higher CK favors growth maintenance, whereas reduced CK may support survival under severe stress [77,78]
Gibberellins (GAs)	Growth restraint under drought, salinity, cold, and pathogen challenge	GA20ox/GA3ox/GA2ox enzymes; GID1 receptors; DELLA proteins	Control growth promotion and integrate environmental constraints with developmental progression	Stem elongation control, seed germination modulation, DELLA accumulation under stress, interaction with defense pathways	GA suppression and DELLA stabilization often favor stress survival by reducing growth expenditure [79]
Brassinosteroids (BRs)	Heat, cold, salinity, drought, oxidative stress, pathogen-associated stress	BRI1/BAK1 receptor complex; BIN2; BES1/BZR1 transcriptional regulators	Promote stress tolerance through cell protection, antioxidant regulation, membrane stabilization, and growth adjustment	ROS-scavenging enzyme activation, stress-responsive gene expression, improved membrane integrity, developmental resilience	Help buffer the cost of stress by partially sustaining growth while enhancing tolerance mechanisms [80]
Strigolactones (SLs)	Drought, nutrient deficiency, root stress, symbiotic interactions	D27, CCD7, CCD8 biosynthesis enzymes; MAX2 signaling component	Modulate root development, resource allocation, symbiosis, and stress adaptation	Root system remodeling, altered shoot branching, enhanced nutrient foraging, interaction with ABA and auxin pathways	Optimize architectural and metabolic resource allocation under limiting conditions [81]
Gasotransmitters and non-canonical signaling molecules, including NO, H_2_S, melatonin, and peptide signals	Drought, salinity, heat, cold, oxidative stress, pathogen challenge, combined stress	NO/RNS metabolism, DES1-mediated H_2_S production, S-nitrosylation, persulfidation, peptide receptors, melatonin-associated redox pathways	Fine-tune redox balance, hormone sensitivity, protein activity, and stress-responsive transcription	Stomatal regulation, antioxidant adjustment, defense priming, modulation of ABA/SA/JA/ET signaling, stress memory-related responses	Function as modulators of hormone and redox networks rather than classical phytohormones; help adjust stress intensity and reduce excessive trade-off costs [82,83]

**Table 2:** Major transcriptional and post-transcriptional regulators governing plant stress responses. This table summarizes the principal regulatory modules that shape stress-responsive gene expression in plants. It includes transcription factor families, RNA-based regulators, and post-transcriptional control systems that collectively determine the amplitude, timing, and specificity of stress adaptation. Their coordinated activity underlies transcriptional reprogramming, developmental plasticity, cellular protection, and stress memory.

Regulatory Class/Family	Representative Members	Major Upstream Signals	Major Stress Types	Typical Target Genes/Processes	Functional Outcome
AP2/ERF-DREB family	DREB1/CBF, DREB2, ERF1, ERF5	Cold signals, dehydration, ABA-independent stress pathways, ET/JA signaling	Cold, drought, salinity, heat, necrotrophic stress	Dehydration-responsive genes, osmoprotectant synthesis, cold acclimation genes, defense genes	Rapid induction of abiotic and defense-associated transcriptional programs [114,115]
bZIP family	ABF/AREB, HY5, TGA factors	ABA, ROS, redox cues, light-stress integration, SA signaling	Drought, salinity, oxidative stress, pathogen-associated stress	ABA-responsive genes, antioxidant enzymes, PR genes, stress-related metabolic regulators	Fine control of ABA-dependent stress adaptation and redox-responsive transcription [116,117]
NAC family	SNAC1, ANAC019, ANAC072/RD26, ATAF1	ABA, drought, salinity, ROS, senescence signals	Drought, salinity, heat, senescence-associated stress	Cell protection genes, detoxification pathways, senescence-related programs, cell wall remodeling	Enhances stress endurance and reallocates resources toward survival [118]
WRKY family	WRKY33, WRKY40, WRKY53, WRKY70	SA, JA, MAPK cascades, ROS, pathogen recognition	Pathogen stress, drought, salinity, oxidative stress	PR genes, hormone-responsive genes, defense metabolite pathways, ROS regulatory genes	Central regulators of immune signaling and hormone-dependent defense balance [119,120]
MYB family	MYB2, MYB15, MYB44, MYB96	ABA, drought, cold, secondary metabolism cues	Drought, salinity, cold, UV and oxidative stress	Cuticular wax biosynthesis, phenylpropanoid metabolism, stomatal regulation, stress-inducible transcription	Couples metabolic reprogramming with protective structural and biochemical responses [121,122]
bHLH family	ICE1, MYC2, bHLH122	Cold signals, JA signaling, ABA, ROS	Cold, herbivory, drought, salinity	Cold-responsive genes, defense pathways, stomatal behavior, metabolic regulation	Integrates developmental control with stress-specific transcriptional activation [123,124]
Heat shock factors (HSFs)	HSFA1, HSFA2, HSFBs	Heat stress, proteotoxic stress, ROS	Heat, oxidative stress, combined stress	Heat shock proteins, chaperones, proteostasis networks	Preserves protein stability and cellular homeostasis during acute stress [125]
Zinc finger proteins	ZAT10, ZAT12, C2H2-type factors	ROS, ABA, salinity, cold	Oxidative stress, salinity, cold, drought	Antioxidant genes, signaling regulators, stress-inducible transcriptional repressors/activators	Fine-tunes signal intensity and prevents excessive cellular damage [126]
MicroRNAs (miRNAs)	miR398, miR156, miR159, miR166, miR393	Stress-triggered transcriptional reprogramming, hormone pathways, ROS	Drought, salinity, heat, cold, pathogen stress	mRNA cleavage or translational repression of TFs, signaling proteins, developmental regulators	Provides rapid post-transcriptional adjustment and improves response precision [127]
Long non-coding RNAs (lncRNAs)	Stress-induced lncRNAs with species-specific functions	Chromatin changes, stress signaling, hormonal cues	Abiotic and biotic stresses, especially combined stresses	Regulation of neighboring genes, miRNA decoy activity, chromatin interaction, transcriptional modulation	Adds regulatory specificity and network plasticity, though many mechanisms remain unresolved [128]
Alternative splicing machinery	SR proteins, spliceosomal regulators, stress-responsive splicing factors	Temperature variation, ABA, ROS, developmental state	Heat, cold, salinity, drought	Isoform switching in signaling kinases, TFs, transporters, and metabolic genes	Expands proteomic and regulatory diversity under fluctuating stress conditions [129]
Epigenetic regulators linked to transcriptional memory	DNA methyltransferases, histone acetyltransferases/ deacetylases, chromatin remodelers	Prolonged or repeated stress, developmental cues	Stress memory, priming, recurrent drought, heat, pathogen challenge	Chromatin accessibility, transcriptional priming, persistent stress-responsive states	Supports short- or long-term stress memory and adaptive recall [130]
RNA decay and translational control factors	RNA-binding proteins, decapping factors, stress granule-associated proteins	Energy limitation, oxidative stress, heat, combined stress	Acute stress and recovery phases	mRNA stability, selective translation, transcript storage or degradation	Helps prioritize essential stress proteins while minimizing unnecessary energy expenditure [131]

**Table 3:** Omics and systems biology approaches advancing mechanistic and translational plant stress research. This table outlines the major analytical platforms currently used to decode plant stress responses across molecular and phenotypic scales. Together, these approaches have enabled a transition from single-gene descriptions toward integrated, predictive, and translational stress biology. Their value lies not only in cataloging stress-responsive components but also in uncovering regulatory hierarchies, biomarkers, candidate genes, and trait-linked pathways for crop improvement.

Approach	Biological Level Captured	Major Insights Generated	Relevance to Stress Biology	Translational Application	Major Limitations
Genomics	DNA sequence variation, structural variants, gene families	Identification of stress-related loci, gene family expansion, allelic diversity, evolutionary adaptation	Reveals the genetic basis of tolerance potential and natural variation	Marker development, QTL mapping, genomic selection, candidate gene discovery	Sequence variation does not directly explain regulatory dynamics or stress-state specificity [160]
Transcriptomics (bulk RNA-seq)	Genome-wide gene expression changes	Stress-responsive genes, pathway activation, co-expression modules, regulatory network inference	Core tool for mapping transcriptional reprogramming under stress	Biomarker discovery, candidate TF identification, comparative stress profiling	Expression changes may not reflect protein activity or cell-type specificity [161]
Single-cell and spatial transcriptomics	Cell-specific and tissue-resolved gene expression	Cellular heterogeneity, tissue-specific signaling, spatially restricted stress responses	Crucial for resolving how stress responses differ across organs and cell	Precision targeting of tissue-specific tolerance traits	High cost, technical complexity, limited coverage in many crop species [162]
Proteomics	Protein abundance, modification, turnover	Post-transcriptional regulation, enzyme dynamics, signaling protein accumulation, stress-induced proteome remodeling	Bridges the gap between transcriptional changes and functional execution	Identification of protein biomarkers, stress-responsive enzymes, pathway bottlenecks	Lower coverage than transcriptomics and difficulty detecting low-abundance regulators [163]
Phosphoproteomics and PTM profiling	Protein phosphorylation and other post-translational modifications	Kinase signaling networks, activation states, rapid signal transduction events	Highly relevant for early stress signaling and pathway activation status	Discovery of actionable regulatory nodes in signaling cascades	Dynamic and technically demanding; often requires precise sampling windows [164]
Metabolomics	Primary and secondary metabolites	Osmoprotectant accumulation, antioxidant metabolites, defense compounds, pathway rewiring	Directly reflects physiological adaptation and stress outcome states	Metabolic biomarkers, quality traits, stress-resilient chemotypes	Strong environmental sensitivity and complex metabolite annotation [165]
Epigenomics	DNA methylation, histone marks, chromatin accessibility	Stress memory, transcriptional priming, chromatin-based regulation	Important for repeated stress exposure and adaptive plasticity	Epigenetic markers and stress priming strategies	Context dependency and unclear stability across generations in many systems [166]
Small RNA profiling	miRNAs, siRNAs, other regulatory RNAs	Post-transcriptional repression networks, fine-tuning of stress pathways	Clarifies how plants rapidly modulate stress gene output	RNA-based biomarkers and regulatory node identification	Functional validation remains slow and mechanistic interpretation can be difficult [167]
Interactomics/network biology	Protein–protein, protein–DNA, gene–gene interactions	Regulatory hubs, signaling crosstalk, network topology, pathway convergence	Essential for understanding integrated stress regulation rather than isolated genes	Prioritization of master regulators for breeding or engineering	Networks are often inferred and require extensive experimental validation [168]
Phenomics	High-throughput morphological, physiological, and imaging traits	Dynamic stress phenotypes, growth responses, recovery kinetics, genotype-by-environment effects	Connects molecular findings to whole-plant performance	Screening elite lines under controlled and semi-field conditions	Trait interpretation can be complex without multi-omics integration [169]
Systems biology and predictive modeling	Multi-layer integration across omics and phenotype	Causal inference, network hierarchy, emergent properties, stress prediction	Enables transition from descriptive to predictive plant stress biology	Decision support for engineering and breeding climate-resilient crops	Model quality depends heavily on data completeness and standardization [170]
Genome editing and functional genomics integration	Targeted gene perturbation and validation	Direct testing of gene function and regulatory hierarchy	Critical for validating omics-derived candidate genes	CRISPR-based development of stress-resilient germplasm	Regulatory constraints, off-target considerations, and polygenic trait complexity [171]
AI-assisted multi-omics analysis	Pattern detection across large heterogeneous datasets	Hidden trait associations, candidate prioritization, predictive stress classification	Powerful for complex, multivariate stress biology datasets	Smart breeding pipelines, predictive phenotyping, precision agriculture integration	Requires large, high-quality datasets and may suffer from poor interpretability [172]

## Data Availability

No new data generated in this study. Data sharing is not applicable to this manuscript.

## References

[1] Karalija E., Ibragić S., Dahija S., Šamec D. (2025). Transgenerational memory of phenotypic traits in plants: Epigenetic regulation of growth, hormonal balance, and stress adaptation. Curr. Issues Mol. Biol..

[2] Siddique A.B., Parveen S., Rahman M.Z., Rahman J. (2024). Revisiting plant stress memory: Mechanisms and contribution to stress adaptation. Physiol. Mol. Biol. Plants.

[3] Moustaka J., Moustakas M. (2023). Early-stage detection of biotic and abiotic stress on plants by chlorophyll fluorescence imaging analysis. Biosensors.

[4] Collins C.G., Elmendorf S.C., Smith J.G., Shoemaker L., Szojka M., Swift M., Suding K.N. (2022). Global change re-structures alpine plant communities through interacting abiotic and biotic effects. Ecol. Lett..

[5] Mittal U., Kumar V., Kukreja S., Singh B., Pandey N.K., Goutam U. (2023). Role of beneficial elements in developing resilience to abiotic and biotic stresses in plants: Present status and future prospects. J. Plant Growth Regul..

[6] Zafar S., Khan M.K., Aslam N., Hasnain Z. (2024). Impact of different stresses on morphology, physiology, and biochemistry of plants. Molecular Dynamics of Plant Stress and Its Management.

[7] Ma L., Li X., Zhang J., Yi D., Li F., Wen H., Liu W., Wang X. (2023). MsWRKY33 increases alfalfa (*Medicago sativa* L.) salt stress tolerance through altering the ROS scavenger via activating MsERF5 transcription. Plant Cell Environ..

[8] Singh A.K., Pal P., Sahoo U.K., Sharma L., Pandey B., Prakash A., Sarangi P.K., Prus P., Pașcalău R., Imbrea F. (2024). Enhancing crop resilience: The role of plant genetics, transcription factors, and next-generation sequencing in addressing salt stress. Int. J. Mol. Sci..

[9] Houetohossou S.C.A., Houndji V.R., Hounmenou C.G., Sikirou R., Kakaï R.L.G. (2023). Deep learning methods for biotic and abiotic stresses detection and classification in fruits and vegetables: State of the art and perspectives. Artif. Intell. Agric..

[10] Ali S., Akhtar M.S., Siraj M., Zaman W. (2024). Molecular communication of microbial plant biostimulants in the rhizosphere under abiotic stress conditions. Int. J. Mol. Sci..

[11] Gull A., Lone A.A., Wani N.U.I. (2019). Biotic and abiotic stresses in plants. Abiotic and Biotic Stress in Plants.

[12] Dangi A.K., Sharma B., Khangwal I., Shukla P. (2018). Combinatorial interactions of biotic and abiotic stresses in plants and their molecular mechanisms: Systems biology approach. Mol. Biotechnol..

[13] Jalmi S.K., Sinha A.K. (2015). ROS mediated MAPK signaling in abiotic and biotic stress-striking similarities and differences. Front. Plant Sci..

[14] Khan A., Shah S.T., Basit A., Mohamed H.I., Li Y. (2024). Mitogen-activated protein kinase: A potent signaling protein that combats biotic and abiotic stress in plants. J. Plant Growth Regul..

[15] Abed M.M., Aydin M., Yiğider E., Ekinci M., Yildirim E. (2025). Systematic Literature Review for Mechanisms and Costs of Plant Adaptation to Biotic and Abiotic Stresses. Phyton (0031-9457).

[16] Ali S., Moon Y.-S., Hamayun M., Khan M.A., Bibi K., Lee I.-J. (2022). Pragmatic role of microbial plant biostimulants in abiotic stress relief in crop plants. J. Plant Interact..

[17] He M., He C.-Q., Ding N.-Z. (2018). Abiotic stresses: General defenses of land plants and chances for engineering multistress tolerance. Front. Plant Sci..

[18] Ramu V.S., Paramanantham A., Ramegowda V., Mohan-Raju B., Udayakumar M., Senthil-Kumar M. (2016). Transcriptome analysis of sunflower genotypes with contrasting oxidative stress tolerance reveals individual-and combined-biotic and abiotic stress tolerance mechanisms. PLoS ONE.

[19] Ku Y.-S., Sintaha M., Cheung M.-Y., Lam H.-M. (2018). Plant hormone signaling crosstalks between biotic and abiotic stress responses. Int. J. Mol. Sci..

[20] Forcat S., Bennett M.H., Mansfield J.W., Grant M.R. (2008). A rapid and robust method for simultaneously measuring changes in the phytohormones ABA, JA and SA in plants following biotic and abiotic stress. Plant Methods.

[21] Singh D., Dhiman V.K., Pandey H., Dhiman V.K., Pandey D. (2022). Crosstalk between salicylic acid and auxins, cytokinins and gibberellins under biotic stress. Auxins, Cytokinins and Gibberellins Signaling in Plants.

[22] Liu T., Chen T., Kan J., Yao Y., Guo D., Yang Y., Ling X., Wang J., Zhang B. (2022). The GhMYB36 transcription factor confers resistance to biotic and abiotic stress by enhancing PR1 gene expression in plants. Plant Biotechnol. J..

[23] Yoon Y., Seo D.H., Shin H., Kim H.J., Kim C.M., Jang G. (2020). The role of stress-responsive transcription factors in modulating abiotic stress tolerance in plants. Agronomy.

[24] Meier S., Bastian R., Donaldson L., Murray S., Bajic V., Gehring C. (2008). Co-expression and promoter content analyses assign a role in biotic and abiotic stress responses to plant natriuretic peptides. BMC Plant Biol..

[25] Chand Jha U., Nayyar H., Mantri N., Siddique K.H.M. (2021). Non-coding RNAs in legumes: Their emerging roles in regulating biotic/abiotic stress responses and plant growth and development. Cells.

[26] Halder K., Chaudhuri A., Abdin M.Z., Majee M., Datta A. (2022). Chromatin-based transcriptional reprogramming in plants under abiotic stresses. Plants.

[27] Bharath P., Gahir S., Raghavendra A.S. (2021). Abscisic acid-induced stomatal closure: An important component of plant defense against abiotic and biotic stress. Front. Plant Sci..

[28] Dietz K.-J., Vogelsang L. (2024). A general concept of quantitative abiotic stress sensing. Trends Plant Sci..

[29] Dopp I.J., Yang X., Mackenzie S.A. (2021). A new take on organelle-mediated stress sensing in plants. New Phytol..

[30] Wang C.-F., Han G.-L., Yang Z.-R., Li Y.-X., Wang B.-S. (2022). Plant salinity sensors: Current understanding and future directions. Front. Plant Sci..

[31] Xu T., Niu J., Jiang Z. (2022). Sensing mechanisms: Calcium signaling mediated abiotic stress in plants. Front. Plant Sci..

[32] Gong Z., Xiong L., Shi H., Yang S., Herrera-Estrella L.R., Xu G., Chao D.-Y., Li J., Wang P.-Y., Qin F. (2020). Plant abiotic stress response and nutrient use efficiency. Sci. China Life Sci..

[33] Shajar F., Nabi A.U., Manzoor A., Saleem S., Mushtaq N.U., Manzoor S., Rehman R.U. (2025). Membrane-associated mechanisms in plant responses to abiotic stress. Plant Sci..

[34] Rodas-Junco B.A., Racagni-Di-Palma G.E., Canul-Chan M., Usorach J., Hernández-Sotomayor S.T. (2021). Link between lipid second messengers and osmotic stress in plants. Int. J. Mol. Sci..

[35] Liang X., Zhang J. (2022). Regulation of plant responses to biotic and abiotic stress by receptor-like cytoplasmic kinases. Stress Biol..

[36] Jin S., Zhong X., Hu Z., Jiang Z. (2025). Ca^2+^ flux in plant responses to abiotic stress. J. Plant Physiol..

[37] Hayes S., Schachtschabel J., Mishkind M., Munnik T., Arisz S.A. (2021). Hot topic: Thermosensing in plants. Plant Cell Environ..

[38] Casal J.J., Murcia G., Bianchimano L. (2024). Plant thermosensors. Annu. Rev. Genet..

[39] Boubakri H. (2026). Microbial-derived inducers of plant immunity: Recent advances and future prospects. Planta.

[40] Nabi Z., Manzoor S., Nabi S.U., Wani T.A., Gulzar H., Farooq M., Arya V.M., Baloch F.S., Vlădulescu C., Popescu S.M. (2024). Pattern-Triggered Immunity and Effector-Triggered Immunity: Crosstalk and cooperation of PRR and NLR-mediated plant defense pathways during host–pathogen interactions. Physiol. Mol. Biol. Plants.

[41] Yadav M., Singh A. (2025). Interplay of calcium sensors with ROS: Unravelling the crosstalk in plant defense response. J. Plant Growth Regul..

[42] Tikhonova O.A., Grigorchuk V.P., Brodovskaya E.V., Veremeichik G.N. (2025). Differential Modulation of Brassinosteroid and Ethylene Signalling Systems by Native and Constitutively Active Forms of the AtCPK1 Gene in Transgenic Tobacco Plants Under Heat Stress. Plants.

[43] Weralupitiya C., Eccersall S., Meisrimler C.-N. (2024). Shared signals, different fates: Calcium and ROS in plant PRR and NLR immunity. Cell Rep..

[44] Peláez-Vico M.Á., Fichman Y., Zandalinas S.I., Foyer C.H., Mittler R. (2024). ROS are universal cell-to-cell stress signals. Curr. Opin. Plant Biol..

[45] Wurzinger B., Mair A., Pfister B., Teige M. (2011). Cross-talk of calcium-dependent protein kinase and MAP kinase signaling. Plant Signal. Behav..

[46] Mata-Pérez C., Sánchez-Vicente I., Arteaga N., Gómez-Jiménez S., Fuentes-Terrón A., Oulebsir C.S., Calvo-Polanco M., Oliver C., Lorenzo Ó. (2023). Functions of nitric oxide-mediated post-translational modifications under abiotic stress. Front. Plant Sci..

[47] Chen S., Jia H., Wang X., Shi C., Wang X., Ma P., Wang J., Ren M., Li J. (2020). Hydrogen sulfide positively regulates abscisic acid signaling through persulfidation of SnRK2. 6 in guard cells. Mol. Plant.

[48] Bihani S.C., Srivastava A.K. (2025). Decoding the calcium signal: Structural insights into CBL-CIPK pathway in plants. Biochim. Et Biophys. Acta (BBA)-Gen. Subj..

[49] Waadt R. (2020). Phytohormone signaling mechanisms and genetic methods for their modulation and detection. Curr. Opin. Plant Biol..

[50] Lin Z., Li Y., Wang Y., Liu X., Ma L., Zhang Z., Mu C., Zhang Y., Peng L., Xie S. (2021). Initiation and amplification of SnRK2 activation in abscisic acid signaling. Nat. Commun..

[51] Zhang Y., Zhou Y., Zhu W., Liu J., Cheng F. (2022). Non-coding RNAs fine-tune the balance between plant growth and abiotic stress tolerance. Front. Plant Sci..

[52] Lim G.-H., Liu H., Yu K., Liu R., Shine M., Fernandez J., Burch-Smith T., Mobley J.K., McLetchie N., Kachroo A. (2020). The plant cuticle regulates apoplastic transport of salicylic acid during systemic acquired resistance. Sci. Adv..

[53] Romero-Hernandez G., Martinez M. (2022). Plant kinases in the perception and signaling networks associated with arthropod herbivory. Front. Plant Sci..

[54] Hartman S., Sasidharan R., Voesenek L.A. (2021). The role of ethylene in metabolic acclimations to low oxygen. New Phytol..

[55] Waadt R., Seller C.A., Hsu P.K., Takahashi Y., Munemasa S., Schroeder J.I. (2022). Publisher Correction: Plant hormone regulation of abiotic stress responses. Nat. Rev. Mol. Cell Biol..

[56] Scuffi D., Álvarez C., Laspina N., Gotor C., Lamattina L., García-Mata C. (2014). Hydrogen sulfide generated by L-cysteine desulfhydrase acts upstream of nitric oxide to modulate abscisic acid-dependent stomatal closure. Plant Physiol..

[57] Pande A., Mun B.G., Rahim W., Khan M., Lee D.S., Lee G.M., Al Azzawi T.N.I., Hussain A., Kim C.K., Yun B.W. (2022). Phytohormonal regulation through protein s-nitrosylation under stress. Front. Plant Sci..

[58] Shah N., Irshad M., Murad W., Hamayun M., Qadir M., Hussain A., Begum H.A., Alrefaei A.F., Almutairi M.H., Ahmad A. (2024). IAA is more effective than EDTA in enhancing phytoremediation potential for cadmium and copper contaminated soils. BMC Plant Biol..

[59] Zhang K., Khan M.N., Khan Z., Luo T., Zhang B., Bi J., Hu L., Luo L. (2024). Seed priming with ascorbic acid and spermidine regulated auxin biosynthesis to promote root growth of rice under drought stress. Front. Plant Sci..

[60] Dini-Andreote F., Wells D.M., Atkinson J.A., Atkinson B.S., Finkel O.M., Castrillo G. (2025). Microbial drivers of root plasticity. New Phytol..

[61] Savelieva E.M., Myakushina Y.A., Lomin S.N., Kolachevskaya O.O., Arkhipov D.V., Romanov G.A. (2026). Biotechnological modification of the cytokinin regulatory system to improve drought and heat tolerance in the major crops. Plant Cell Rep..

[62] Zhang R., Wang Y., Wang X., Jiao S., Lu Y., Du Y., Zhang W., Kang Y., Liu Y., Qin S. (2025). Differential responses of microstructure, antioxidant defense, and plant hormone signaling regulation in potato (*Solanum tuberosum* L.) under drought, alkaline salt, and combined stresses. Sci. Hortic..

[63] Guo S.-Q., Chen Y.-X., Ju Y.-L., Pan C.-Y., Shan J.-X., Ye W.-W., Dong N.-Q., Kan Y., Yang Y.-B., Zhao H.-Y. (2025). Fine-tuning gibberellin improves rice alkali–thermal tolerance and yield. Nature.

[64] Wu X., Li L., Hannan F., Qin T., Ayyaz A., Ma J., Athar H.U.R., Zafar Z.U., Farooq M.A., Zhou W. (2024). Brassinosteroid-induced transcriptomic rearrangements unveiled the physiological mechanism of chromium stress tolerance in Brassica napus. Curr. Plant Biol..

[65] Sang S., Tian S., Xu W., Shi H., Deng B. (2025). Dual defense: Melatonin simultaneously mitigates cadmium toxicity and southern blight in peanut. Front. Plant Sci..

[66] Jariani P., Sabokdast M., Rajabi F., Naghavi M.R., Dedicova B. (2025). Enhancing salinity tolerance in wheat: The role of synthetic Strigolactone (GR24) in modulating antioxidant enzyme activities, ion channels, and related gene expression in stress responses. Sci. Rep..

[67] Shen J., Tong M., Yuan Q., Long L., Shi Y. (2025). Exogenous strigolactones modulate antioxidant metabolism via CsD27 to enhance drought tolerance in tea plants. Front. Plant Sci..

[68] Ali S., Mir R.A., Haque M.A., Danishuddin, Almalki M.A., Alfredan M., Khalifa A., Mahmoudi H., Shahid M., Tyagi A. (2025). Exploring physiological and molecular dynamics of drought stress responses in plants: Challenges and future directions. Front. Plant Sci..

[69] An S., Liu Y., Sang K., Wang T., Yu J., Zhou Y., Xia X. (2023). Brassinosteroid signaling positively regulates abscisic acid biosynthesis in response to chilling stress in tomato. J. Integr. Plant Biol..

[70] Zavaliev R., Dong X. (2024). NPR1, a key immune regulator for plant survival under biotic and abiotic stresses. Mol. Cell.

[71] Wu Y., Zhang D., Chu J.Y., Boyle P., Wang Y., Brindle I.D., De Luca V., Després C. (2012). The Arabidopsis NPR1 protein is a receptor for the plant defense hormone salicylic acid. Cell Rep..

[72] Zhu Z. (2023). Jasmonate: The Swiss army knife in the plant’s pocket. J. Exp. Bot..

[73] Ma Z., Hu L., Jiang W. (2024). Understanding AP2/ERF transcription factor responses and tolerance to various abiotic stresses in plants: A comprehensive review. Int. J. Mol. Sci..

[74] Khan S., Alvi A.F., Khan N.A. (2024). Role of ethylene in the regulation of plant developmental processes. Stresses.

[75] Gao J., Zhuang S., Zhang W. (2024). Advances in plant auxin biology: Synthesis, metabolism, signaling, interaction with other hormones, and roles under abiotic stress. Plants.

[76] Leftley N., Banda J., Pandey B., Bennett M., Voß U. (2021). Uncovering how auxin optimizes root systems architecture in response to environmental stresses. Cold Spring Harb. Perspect. Biol..

[77] Hajam A.H., Ali M.S., Singh S.K., Bashri G. (2024). Understanding cytokinin: Biosynthesis, signal transduction, growth regulation, and phytohormonal crosstalk under heavy metal stress. Environ. Exp. Bot..

[78] Mason M.G., Jha D., Salt D.E., Tester M., Hill K., Kieber J.J., Eric Schaller G. (2010). Type-B response regulators ARR1 and ARR12 regulate expression of AtHKT1; 1 and accumulation of sodium in Arabidopsis shoots. Plant J..

[79] Bouré N., Arnaud N. (2023). Molecular GA pathways as conserved integrators for adaptive responses. Plant Biol..

[80] Yin W., Dong N., Li X., Yang Y., Lu Z., Zhou W., Qian Q., Chu C., Tong H. (2025). Understanding brassinosteroid-centric phytohormone interactions for crop improvement. J. Integr. Plant Biol..

[81] Khalid M.F., Shafqat W., Khan R.I., Jawaid M.Z., Hussain S., Saqib M., Rizwan M., Ahmed T. (2024). Unveiling the resilience mechanism: Strigolactones as master regulators of plant responses to abiotic stresses. Plant Stress.

[82] Datta T., Kumar R.S., Sinha H., Trivedi P.K. (2024). Small but mighty: Peptides regulating abiotic stress responses in plants. Plant Cell Environ..

[83] Khanna K., Sharma N., Kour S., Ali M., Ohri P., Bhardwaj R. (2021). Hydrogen sulfide: A robust combatant against abiotic stresses in plants. Hydrogen.

[84] Veselova S., Nuzhnaya T., Burkhanova G., Rumyantsev S., Maksimov I. (2026). Wheat miR408 and miR159 Weaken the Virulence of Parastagonospora nodorum (Berk.) and Induce the Defense Response in Plants (*Triticum aestivum* L.) Against Pathogens. Plants.

[85] Zhou H., Hua J., Zhang J., Luo S. (2022). Negative interactions balance growth and defense in plants confronted with herbivores or pathogens. J. Agric. Food Chem..

[86] Seo J.S., Um T. (2025). Modulation of JA–GA signaling and drought response by a truncated OsJAZ9 protein in rice. Plant Signal. Behav..

[87] Mandal D., Datta S., Mitra S., Nag Chaudhuri R. (2024). ABI3 promotes auxin signalling by regulating SHY2 expression to control primary root growth in response to dehydration stress. J. Exp. Bot..

[88] Kosakivska I., Voytenko L., Vasyuk V., Shcherbatiuk M. (2024). ABA-induced alterations in cytokinin homeostasis of Triticum aestivum and Triticum spelta under heat stress. Plant Stress.

[89] Neres D.F., Wright R.C. (2024). Pleiotropy, a feature or a bug? Toward co-ordinating plant growth, development, and environmental responses through engineering plant hormone signaling. Curr. Opin. Biotechnol..

[90] Figueroa-Macías J.P., García Y.C., Núñez M., Díaz K., Olea A.F., Espinoza L. (2021). Plant growth-defense trade-offs: Molecular processes leading to physiological changes. Int. J. Mol. Sci..

[91] Wu X., Zhu S., He L., Cheng G., Li T., Meng W., Wen F. (2025). Phenylalanine Ammonia-Lyase: A Core Regulator of Plant Carbon Metabolic Flux Redistribution-From Molecular Mechanisms and Growth Modulation to Stress Adaptability. Plants.

[92] Cowan A.K., Freeman M., Björkman P.-O., Nicander B., Sitbon F., Tillberg E. (2005). Effects of senescence-induced alteration in cytokinin metabolism on source-sink relationships and ontogenic and stress-induced transitions in tobacco. Planta.

[93] Aerts N., Pereira Mendes M., Van Wees S.C. (2021). Multiple levels of crosstalk in hormone networks regulating plant defense. Plant J..

[94] Altaf M., Mahmood S.R., Ahmad O., Bashir S., Bashir A., Showkat S., Aijaz B., Majid B. (2025). The Emerging Role of Phytohormone Crosstalk in Orchestrating Plant Stress Tolerance. Plant Cell Biotechnol. Mol. Biol..

[95] Khan S. (2016). Identification and Prioritization of Dermatological Disorders Influenced by Female Hormonal Fluctuations During and Beyond Menopause.

[96] Cao M.-J., Zhang Y.-L., Liu X., Huang H., Zhou X.E., Wang W.-L., Zeng A., Zhao C.-Z., Si T., Du J. (2017). Combining chemical and genetic approaches to increase drought resistance in plants. Nat. Commun..

[97] Wang K., Guo H., Yin Y. (2024). AP2/ERF transcription factors and their functions in Arabidopsis responses to abiotic stresses. Environ. Exp. Bot..

[98] Bhoite R., Onyemaobi O., Halder T., Shankar M., Sharma D. (2025). Transcription factors–Insights into abiotic and biotic stress resilience and crop improvement. Curr. Plant Biol..

[99] Zha D., He Y., Song J. (2025). Regulatory role of ABA-responsive element binding factors in plant abiotic stress response. Physiol. Plant..

[100] Yang Y., Xu Y., Feng B., Li P., Li C., Zhu C.-Y., Ren S.-N., Wang H.-L. (2025). Regulatory networks of bZIPs in drought, salt and cold stress response and signaling. Plant Sci..

[101] Xiong H., He H., Chang Y., Miao B., Liu Z., Wang Q., Dong F., Xiong L. (2025). Multiple roles of NAC transcription factors in plant development and stress responses. J. Integr. Plant Biol..

[102] Zheng C., Yang Q., Wang X., Chen Y., He R., Li X., Pan H., Zhuo R., Qu T., Qiu W. (2025). Transcription Factors Involved in Plant Stress and Growth and Development: NAC. Agronomy.

[103] Tang Y., Xia P. (2025). WRKY transcription factors: Key regulators in plant drought tolerance. Plant Sci..

[104] Huang Y., Sun Z., Zhou X. (2024). WRKY transcription factors in response to metal stress in plants: A review. Int. J. Mol. Sci..

[105] Rattan P., Gandotra E., Mittal S. (2025). Long non-coding RNAs: Silent contributors to plant survival under abiotic stress. Biochem. Biophys. Res. Commun..

[106] Zhang F., Yang J., Zhang N., Wu J., Si H. (2022). Roles of microRNAs in abiotic stress response and characteristics regulation of plant. Front. Plant Sci..

[107] Urquiaga M.C.d.O., Thiebaut F., Hemerly A.S., Ferreira P.C.G. (2021). From trash to luxury: The potential role of plant LncRNA in DNA methylation during abiotic stress. Front. Plant Sci..

[108] Gelaw T.A., Sanan-Mishra N. (2021). Non-coding RNAs in response to drought stress. Int. J. Mol. Sci..

[109] Liu X.-X., Guo Q.-H., Xu W.-B., Liu P., Yan K. (2022). Rapid regulation of alternative splicing in response to environmental stresses. Front. Plant Sci..

[110] Wu H.-Y.L., Hsu P.Y. (2021). Nonsense-mediated decay is not a major mechanism for regulating the uORF-containing mRNAs in Arabidopsis. bioRxiv.

[111] Maruri-López I., Figueroa N.E., Hernández-Sánchez I.E., Chodasiewicz M. (2021). Plant stress granules: Trends and beyond. Front. Plant Sci..

[112] Jang G.-J., Jang J.-C., Wu S.-H. (2020). Dynamics and functions of stress granules and processing bodies in plants. Plants.

[113] Yao T., Zhang J., Xie M., Yuan G., Tschaplinski T.J., Muchero W., Chen J.-G. (2021). Transcriptional regulation of drought response in Arabidopsis and woody plants. Front. Plant Sci..

[114] Varghese R., Shringi H., Efferth T., Ramamoorthy S. (2025). Artificial intelligence driven approaches in phytochemical research: Trends and prospects. Phytochem. Rev..

[115] Zhao W., Dong H., Cui C., Wang C., Liu Y., Ning Y., Zhang H., Li M., Li S. (2025). Integrated transcriptomic and WGCNA identify drought-responsive TFs in Larix olgensis. BMC Plant Biol..

[116] Zhang Y., Xia P. (2023). The DREB transcription factor, a biomacromolecule, responds to abiotic stress by regulating the expression of stress-related genes. Int. J. Biol. Macromol..

[117] Yoshida T., Fujita Y., Sayama H., Kidokoro S., Maruyama K., Mizoi J., Shinozaki K., Yamaguchi-Shinozaki K. (2010). AREB1, AREB2, and ABF3 are master transcription factors that cooperatively regulate ABRE-dependent ABA signaling involved in drought stress tolerance and require ABA for full activation. Plant J..

[118] Baoxiang W., Zhiguang S., Yan L., Bo X., Jingfang L., Ming C., Yungao X., Bo Y., Jian L., Jinbo L. (2023). A pervasive phosphorylation cascade modulation of plant transcription factors in response to abiotic stress. Planta.

[119] Usman M., Azam M., Song C., Yousaf N., Ahmad Z., Murtaza G., Manzoor M.A. (2025). Integrating biochemical pathways, transcriptional regulation, proteomics, and genetic approaches for enhancing salt stress tolerance in crops. Trop. Plants.

[120] Chen H., Lai Z., Shi J., Xiao Y., Chen Z., Xu X. (2010). Roles of Arabidopsis WRKY18, WRKY40 and WRKY60 transcription factors in plant responses to abscisic acid and abiotic stress. BMC Plant Biol..

[121] Ma Z., Hu L. (2024). WRKY transcription factor responses and tolerance to abiotic stresses in plants. Int. J. Mol. Sci..

[122] Seo PilJoon S.P., Lee SaetBuyl L.S., Suh MiChung S.M., Park MiJeong P.M., Go YoungSam G.Y., Park ChungMo P.C. (2011). The MYB96 transcription factor regulates cuticular wax biosynthesis under drought conditions in Arabidopsis. Plant Cell.

[123] Ma R., Liu B., Geng X., Ding X., Yan N., Sun X., Wang W., Sun X., Zheng C. (2023). Biological function and stress response mechanism of MYB transcription factor family genes. J. Plant Growth Regul..

[124] Chinnusamy V., Ohta M., Kanrar S., Lee B.-H., Hong X., Agarwal M., Zhu J.-K. (2003). ICE1: A regulator of cold-induced transcriptome and freezing tolerance in Arabidopsis. Genes Dev..

[125] Fragkostefanakis S., Mesihovic A., Simm S., Paupière M.J., Hu Y., Paul P., Mishra S.K., Tschiersch B., Theres K., Bovy A. (2016). HsfA2 controls the activity of developmentally and stress-regulated heat stress protection mechanisms in tomato male reproductive tissues. Plant Physiol..

[126] Moulick D., Bhutia K.L., Sarkar S., Roy A., Mishra U.N., Pramanick B., Maitra S., Shankar T., Hazra S., Skalicky M. (2023). The intertwining of Zn-finger motifs and abiotic stress tolerance in plants: Current status and future prospects. Front. Plant Sci..

[127] Rehman S., Ahmad Z., Ramakrishnan M., Kalendar R., Zhuge Q. (2023). Regulation of plant epigenetic memory in response to cold and heat stress: Towards climate resilient agriculture. Funct. Integr. Genom..

[128] Lagiotis G., Madesis P., Stavridou E. (2023). Echoes of a stressful past: Abiotic stress memory in crop plants towards enhanced adaptation. Agriculture.

[129] Matinahmadi A., Zayani Z., Majewska K., Smoliński D.J. (2026). Plant P-bodies in post-transcriptional control: Composition, dynamics, and context-dependent roles. Plant Commun..

[130] Xiang Y., Dong X. (2025). Translational regulation of plant stress responses: Mechanisms, pathways, and applications in bioengineering. Annu. Rev. Phytopathol..

[131] Escalante L.E., Gasch A.P. (2021). The role of stress-activated RNA–protein granules in surviving adversity. RNA.

[132] Kumar V., Wegener M., Knieper M., Kaya A., Viehhauser A., Dietz K.-J. (2024). Strategies of molecular signal integration for optimized plant acclimation to stress combinations. Plant Stress Toler. Methods Protoc..

[133] Tan Q.W., Lim P.K., Chen Z., Pasha A., Provart N., Arend M., Nikoloski Z., Mutwil M. (2023). Cross-stress gene expression atlas of Marchantia polymorpha reveals the hierarchy and regulatory principles of abiotic stress responses. Nat. Commun..

[134] Sheard L.B., Tan X., Mao H., Withers J., Ben-Nissan G., Hinds T.R., Kobayashi Y., Hsu F.-F., Sharon M., Browse J. (2010). Jasmonate perception by inositol-phosphate-potentiated COI1–JAZ co-receptor. Nature.

[135] Pardo-Hernández M., Arbona V., Simon I., Rivero R.M. (2024). Specific ABA-independent tomato transcriptome reprogramming under abiotic stress combination. Plant J..

[136] Jing Z., Liu N., Zhang Z., Hou X. (2024). Research progress on plant responses to stress combinations in the context of climate change. Plants.

[137] Jiang Z., Verhoeven A., Li Y., Geertsma R., Sasidharan R., van Zanten M. (2024). Deciphering acclimation to sublethal combined and sequential abiotic stresses in Arabidopsis thaliana. Plant Physiol..

[138] Ludwig E., Sumner J., Berry J., Polydore S., Ficor T., Agnew E., Haines K., Greenham K., Fahlgren N., Mockler T.C. (2024). Natural variation in Brachypodium distachyon responses to combined abiotic stresses. Plant J..

[139] Balfagón Sanmartín D. (2024). WRKY48 negatively regulates plant acclimation to a combination of high light and heat stress. Sci. Comm..

[140] Pandey P., Irulappan V., Bagavathiannan M.V., Senthil-Kumar M. (2017). Impact of combined abiotic and biotic stresses on plant growth and avenues for crop improvement by exploiting physio-morphological traits. Front. Plant Sci..

[141] Munns R., Millar A.H. (2023). Seven plant capacities to adapt to abiotic stress. J. Exp. Bot..

[142] Xu Y., Fu X. (2022). Reprogramming of plant central metabolism in response to abiotic stresses: A metabolomics view. Int. J. Mol. Sci..

[143] Vera-Vives A.M., Mellon M., Gurrieri L., Westhoff P., Segalla A., Bizzotto E., Campanaro S., Sparla F., Weber A.P., Alboresi A. (2024). Inactivation of mitochondrial complex IV in Physcomitrium patens reveals the essential role of respiration in coordinating plants metabolism. bioRxiv.

[144] Marmagne A., Masclaux-Daubresse C., Chardon F. (2022). Modulation of plant nitrogen remobilization and postflowering nitrogen uptake under environmental stresses. J. Plant Physiol..

[145] Vanlerberghe G.C., Dahal K., Alber N.A., Chadee A. (2020). Photosynthesis, respiration and growth: A carbon and energy balancing act for alternative oxidase. Mitochondrion.

[146] Rodrigues Neto J.C., Salgado F.F., Braga Í.d.O., Carvalho da Silva T.L., Belo Silva V.N., Leão A.P., Ribeiro J.A.d.A., Abdelnur P.V., Valadares L.F., De Sousa C.A.F. (2023). Osmoprotectants play a major role in the Portulaca oleracea resistance to high levels of salinity stress—Insights from a metabolomics and proteomics integrated approach. Front. Plant Sci..

[147] Nicolas P., Shinozaki Y., Powell A., Philippe G., Snyder S.I., Bao K., Zheng Y., Xu Y., Courtney L., Vrebalov J. (2022). Spatiotemporal dynamics of the tomato fruit transcriptome under prolonged water stress. Plant Physiol..

[148] Tran L.-S.P., Nakashima K., Sakuma Y., Simpson S.D., Fujita Y., Maruyama K., Fujita M., Seki M., Shinozaki K., Yamaguchi-Shinozaki K. (2004). Isolation and functional analysis of Arabidopsis stress-inducible NAC transcription factors that bind to a drought-responsive cis-element in the early responsive to dehydration stress 1 promoter. Plant Cell.

[149] Correia P.M., Cairo Westergaard J., Bernardes da Silva A., Roitsch T., Carmo-Silva E., Marques da Silva J. (2022). High-throughput phenotyping of physiological traits for wheat resilience to high temperature and drought stress. J. Exp. Bot..

[150] Bagherian K., Bidese-Puhl R., Bao Y., Zhang Q., Sanz-Saez A., Dang P.M., Lamb M.C., Chen C. (2023). Phenotyping agronomic and physiological traits in peanut under mid-season drought stress using UAV-based hyperspectral imaging and machine learning. Plant Phenome J..

[151] Zahid A., Dashtipour K., Abbas H.T., Mabrouk I.B., Al-Hasan M., Ren A., Imran M.A., Alomainy A., Abbasi Q.H. (2022). Machine learning enabled identification and real-time prediction of living plants’ stress using terahertz waves. Def. Technol..

[152] Heinemann A.B., Costa-Neto G., Fritsche-Neto R., da Matta D.H., Fernandes I.K. (2022). Enviromic prediction is useful to define the limits of climate adaptation: A case study of common bean in Brazil. Field Crops Res..

[153] Cooper M., Tomura S., Wilkinson M.J., Powell O., Messina C.D. (2025). Breeding perspectives on tackling trait genome-to-phenome (g2p) dimensionality using ensemble-based genomic prediction. Theor. Appl. Genet..

[154] Smith D.T., Potgieter A.B., Chapman S.C. (2021). Scaling up high-throughput phenotyping for abiotic stress selection in the field. Theor. Appl. Genet..

[155] Jin J., Cheng L., Meng L., Su H., Lu P., Tao J., Zhang W., Liu N., Li H., Zhang J. (2024). Single-nucleus RNA sequencing of Nicotiana tabacum seedlings reveals heterogeneity among cell types. Ind. Crops Prod..

[156] Asmat P., Hidalgo M. (2025). From Rhizosphere to Crop Production: Evolutionary and Molecular Insights into Plant Growth Promoting Rhizobacteria (PGPR). Ann. Agri-Bio Res..

[157] Krejci A., Tennessen J.M. (2017). Metabolism in time and space–exploring the frontier of developmental biology. Development.

[158] Kundu B.K., Tanti B. (2026). Decoding plant physiology through systems biology: Integrative multi-omics and computational perspectives for next-generation crop design. Plant Commun..

[159] Pagnotta M.A. (2025). Molecular breeding for abiotic stress tolerance in crops: Recent developments and future prospectives. Int. J. Mol. Sci..

[160] Kahlon K.S., Rawale K.S., Kumar S., Gill K.S. (2024). Identification and mapping of QTLs and their corresponding candidate genes controlling high night-time temperature stress tolerance in wheat (*Triticum aestivum* L.). Plant Genome.

[161] Panahi B., Shahi A. (2024). Trancriptome data mining in combination with co-expression network analysis identifies the functional modules and critical regulators in *Hordeum vulgare* L. in response to cold stress. Biochem. Biophys. Rep..

[162] Sun X., Feng D., Liu M., Qin R., Li Y., Lu Y., Zhang X., Wang Y., Shen S., Ma W. (2022). Single-cell transcriptome reveals dominant subgenome expression and transcriptional response to heat stress in Chinese cabbage. Genome Biol..

[163] Habibpourmehraban F., Atwell B.J., Haynes P.A. (2022). Unique and shared proteome responses of rice plants (Oryza sativa) to individual abiotic stresses. Int. J. Mol. Sci..

[164] Li Q., Sun Q., Wang D., Liu Y., Zhang P., Lu H., Zhang Y., Zhang S., Wang A., Ding X. (2022). Quantitative phosphoproteomics reveals the role of wild soybean GsSnRK1 as a metabolic regulator under drought and alkali stresses. J. Proteom..

[165] Wang J., Yang K., Yao L., Ma Z., Li C., Si E., Li B., Meng Y., Ma X., Shang X. (2021). Metabolomics analyses provide insights into nutritional value and abiotic stress tolerance in halophyte Halogeton glomeratus. Front. Plant Sci..

[166] Kou S., Gu Q., Duan L., Liu G., Yuan P., Li H., Wu Z., Liu W., Huang P., Liu L. (2022). Genome-wide bisulphite sequencing uncovered the contribution of DNA methylation to rice short-term drought memory formation. J. Plant Growth Regul..

[167] Öztürk Gökçe Z.N., Aksoy E., Bakhsh A., Demirel U., Çalışkan S., Çalışkan M.E. (2021). Combined drought and heat stresses trigger different sets of miRNAs in contrasting potato cultivars. Funct. Integr. Genom..

[168] Altmann M., Altmann S., Rodriguez P.A., Weller B., Vergara L.E., Palme J., Marín-de la Rosa N. (2020). Extensive signal integration by the phytohormone protein network. Nature.

[169] Joshi S., Thoday-Kennedy E., Daetwyler H.D., Hayden M., Spangenberg G., Kant S. (2021). High-throughput phenotyping to dissect genotypic differences in safflower for drought tolerance. PLoS ONE.

[170] Filippou P., Zarza X., Antoniou C., Obata T., Villarroel C.A., Ganopoulos I., Harokopos V., Gohari G., Aidinis V., Madesis P. (2021). Systems biology reveals key tissue-specific metabolic and transcriptional signatures involved in the response of Medicago truncatula plant genotypes to salt stress. Comput. Struct. Biotechnol. J..

[171] Hazbir N.A.M., Jumali S.S., Shakri T., Isa N.M. (2023). Characterization and functional study of stress-associated protein in rice and Arabidopsis. Malays. Appl. Biol..

[172] Murmu S., Sinha D., Chaurasia H., Sharma S., Das R., Jha G.K., Archak S. (2024). A review of artificial intelligence-assisted omics techniques in plant defense: Current trends and future directions. Front. Plant Sci..

[173] Husaini A.M. (2022). High-value pleiotropic genes for developing multiple stress-tolerant biofortified crops for 21st-century challenges. Heredity.

[174] Abraham-Juárez M.J., Busche M., Anderson A.A., Lunde C., Winders J., Christensen S.A., Hunter C.T., Hake S., Brunkard J.O. (2022). Liguleless narrow and narrow odd dwarf act in overlapping pathways to regulate maize development and metabolism. Plant J..

[175] Singh A., Maurya A., Rajkumar S., Singh A.K., Bhardwaj R., Kaushik S.K., Kumar S., Singh K., Singh G.P., Singh R. (2024). Genome-wide comparative analysis of five Amaranthaceae species reveals a large amount of repeat content. Plants.

[176] Tan J.W., Shinde H., Tesfamicael K., Hu Y., Fruzangohar M., Tricker P., Baumann U., Edwards E.J., Rodríguez López C.M. (2023). Global transcriptome and gene co-expression network analyses reveal regulatory and non-additive effects of drought and heat stress in grapevine. Front. Plant Sci..

[177] Yan Z., Wang J., Wang F., Xie C., Lv B., Yu Z., Dai S., Liu X., Xia G., Tian H. (2021). MPK3/6-induced degradation of ARR1/10/12 promotes salt tolerance in Arabidopsis. EMBO Rep..

[178] Ojolo S.P., Cao S., Priyadarshani S., Li W., Yan M., Aslam M., Zhao H., Qin Y. (2018). Regulation of plant growth and development: A review from a chromatin remodeling perspective. Front. Plant Sci..

[179] Wei X., Liu S., Sun C., Xie G., Wang L. (2021). Convergence and divergence: Signal perception and transduction mechanisms of cold stress in Arabidopsis and rice. Plants.

[180] Cheng K.C., Burdine R.D., Dickinson M.E., Ekker S.C., Lin A.Y., Lloyd K.K., Lutz C.M., MacRae C.A., Morrison J.H., O’Connor D.H. (2022). Promoting validation and cross-phylogenetic integration in model organism research. Dis. Models Mech..

[181] Dezem F.S., Arjumand W., DuBose H., Morosini N.S., Plummer J. (2024). Spatially resolved single-cell omics: Methods, challenges, and future perspectives. Annu. Rev. Biomed. Data Sci..

[182] Faye J.M., Akata E.A., Sine B., Diatta C., Cisse N., Fonceka D., Morris G.P. (2022). Quantitative and population genomics suggest a broad role of stay-green loci in the drought adaptation of sorghum. Plant Genome.

[183] Faist H., Trognitz F., Antonielli L., Symanczik S., White P.J., Sessitsch A. (2023). Potato root-associated microbiomes adapt to combined water and nutrient limitation and have a plant genotype-specific role for plant stress mitigation. Environ. Microbiome.

[184] Lumibao C.Y., Torres Martínez L., Megonigal J.P., Van Bael S.A., Blum M.J. (2022). Microbial mediation of salinity stress response varies by plant genotype and provenance over time. Mol. Ecol..

[185] Barua D., Mishra A., Kirti P., Barah P. (2022). Identifying signal-crosstalk mechanism in maize plants during combined salinity and boron stress using integrative systems biology approaches. BioMed Res. Int..

[186] Morales A., De Boer H.J., Douma J.C., Elsen S., Engels S., Glimmerveen T., Sajeev N., Huber M., Luimes M., Luitjens E. (2022). Effects of sublethal single, simultaneous and sequential abiotic stresses on phenotypic traits of Arabidopsis thaliana. AoB Plants.

[187] Ramkumar M., Mulani E., Jadon V., Sureshkumar V., Krishnan S.G., Senthil Kumar S., Raveendran M., Singh A., Solanke A.U., Singh N. (2022). Identification of major candidate genes for multiple abiotic stress tolerance at seedling stage by network analysis and their validation by expression profiling in rice (*Oryza sativa* L.). 3 Biotech.

[188] Lauterberg M., Saranga Y., Deblieck M., Klukas C., Krugman T., Perovic D., Ordon F., Graner A., Neumann K. (2022). Precision phenotyping across the life cycle to validate and decipher drought-adaptive QTLs of wild emmer wheat (*Triticum turgidum* ssp. dicoccoides) introduced into elite wheat varieties. Front. Plant Sci..

[189] Pardo J., Wai C.M., Harman M., Nguyen A., Kremling K.A., Romay M.C., Lepak N., Bauerle T.L., Buckler E.S., Thompson A.M. (2023). Cross-species predictive modeling reveals conserved drought responses between maize and sorghum. Proc. Natl. Acad. Sci. USA.

